# Engineered *Escherichia coli*-Derived dsRNA Identifies β-Tubulin as a Candidate RNAi Target in Mosquito Larvae

**DOI:** 10.3390/insects17090880

**Published:** 2026-08-23

**Authors:** Kai Wang, Zhongdan Bi, Teng Zhao, Yuxi Pan, Jing Wu, Xiaohui Liu, Dan Xing, Jiahong Wu, Chunxiao Li

**Affiliations:** 1 Key Laboratory of Environmental Pollution Monitoring and Disease Control, School of Public Health, Ministry of Education, Guizhou Medical University, Guiyang 561113, China; 2 State Key Laboratory of Pathogen and Biosecurity, Beijing 100071, China; 3 Hunan Provincial Key Laboratory of Animal Intestinal Function and Regulation, College of Life Sciences, Hunan Normal University, Changsha 410081, China; 4 Characteristic Key Laboratory of Modern Pathogen Biology, School of Basic Medicine Sciences, Guizhou Medical University, Gui-an New Area 561113, China

**Keywords:** RNAi, engineered bacteria-derived dsRNA, β-tubulin, *Aedes albopictus*, *Culex quinquefasciatus*

## Abstract

Identifying conserved RNAi targets that remain effective across mosquito species and resistance backgrounds is important for developing broadly applicable mosquito control tools. Here, an engineered *Escherichia coli* HT115-L4440 system was used to produce β-tubulin double-stranded RNA (dsRNA), and its larvicidal activity was evaluated in susceptible and pyrethroid-resistant strains of *Culex quinquefasciatus* and *Aedes albopictus*. Recombinant bacteria produced species-specific β-tubulin dsRNA fragments of approximately 360 bp. Immersion treatment caused high larval mortality in both species. In *Cx. quinquefasciatus*, mortality reached 85.00% and 89.00% in susceptible and resistant strains, respectively, compared with 8.00% and 10.00% in controls, while β-tubulin transcript abundance showed maximum reductions of 571-fold and 333-fold, respectively. In *Ae. albopictus*, mortality reached 91.00% and 82.00% in susceptible and resistant strains, compared with 9% and 8% in controls, with maximum reductions in β-tubulin expression of 157-fold and 337-fold, respectively. These comparable effects suggest that β-tubulin dsRNA is not compromised by conventional pyrethroid-resistance mechanisms. Transcriptomic and proteomic analyses showed that β-tubulin knockdown disrupted key cellular processes, including cytoskeletal regulation, metabolism, protein synthesis, intracellular transport, detoxification, oxidative stress, DNA replication, and autophagy. Overall, engineered bacteria-derived β-tubulin dsRNA induced consistent gene silencing and larval mortality in both susceptible and resistant mosquito larvae, supporting β-tubulin as a conserved candidate RNAi target for mosquito control.

## Simple Summary

Mosquitoes transmit many important diseases, and increasing insecticide resistance has reduced the effectiveness of conventional control methods. RNA interference (RNAi) provides a promising alternative by specifically reducing the expression of essential genes. In this study, we used an established *Escherichia coli*-based expression system to produce double-stranded RNA (dsRNA) targeting β-tubulin and evaluated its effects in *Aedes albopictus* and *Culex quinquefasciatus* larvae with different insecticide susceptibility backgrounds. The β-tubulin dsRNA effectively reduced target gene expression and caused high larval mortality in both mosquito species, including pyrethroid-resistant populations. These findings suggest that β-tubulin is a promising RNAi target whose effects are maintained across different mosquito species and insecticide susceptibility backgrounds, providing a potential basis for the development of future RNAi-based mosquito control strategies.

## 1. Introduction

Mosquitoes are important vectors of several human pathogens, and their control remains essential for reducing the transmission of mosquito-borne diseases. However, the long-term and intensive use of chemical insecticides has accelerated the development of insecticide resistance, reducing the effectiveness of conventional vector-control strategies [1,2,3,4,5,6].

RNA interference (RNAi) provides a promising alternative for mosquito control because it enables sequence-specific silencing of essential genes through dsRNA-mediated degradation of target mRNAs. However, its practical application remains constrained by several factors, including the identification of effective target genes, degradation of dsRNA under environmental and digestive conditions, inefficient uptake and delivery into target tissues, and the cost of large-scale dsRNA production [7,8,9,10,11,12,13,14,15,16].

Recent studies have therefore focused on improving dsRNA stability and delivery through oral formulations, nanoparticle-based carriers, and microbial expression or symbiont-mediated delivery systems, highlighting continued progress toward more practical RNAi-based mosquito control strategies [17,18,19,20]. Among available dsRNA production platforms, microbial expression systems provide a relatively cost-effective and scalable approach. *Escherichia coli* HT115 (DE3), an RNase III-deficient strain compatible with the L4440 expression system, has been widely used for dsRNA production and has also been successfully applied to oral RNAi in mosquitoes [21,22].

β-tubulin is a conserved structural protein involved in microtubule formation, cell division, intracellular transport, and cellular homeostasis [23,24]. Its essential role in cellular function, together with previous evidence that β-tubulin silencing can impair mosquito larval development and survival, makes it a promising RNAi target for mosquito control [25]. However, the practical utility of such a target would be strengthened if its RNAi-mediated effects were maintained across different mosquito species and insecticide-resistance backgrounds. Therefore, the present study evaluated β-tubulin RNAi in two mosquito species with different insecticide-susceptibility backgrounds.

In this study, we utilized the established *E. coli* HT115(DE3)-L4440 dsRNA expression system to produce β-tubulin dsRNA and evaluated its potential as an RNAi target for mosquito control. We evaluated the larvicidal activity and gene-silencing efficiency of bacteria-derived β-tubulin dsRNA in both susceptible and pyrethroid-resistant strains and further investigated the molecular responses induced by β-tubulin knockdown using transcriptomic and proteomic analyses. This study aimed to validate β-tubulin as a conserved candidate target gene for RNAi-based mosquito control and to assess the feasibility of engineered bacteria-derived dsRNA as a scalable production strategy.

An engineered *E. coli*-based dsRNA delivery system was developed to silence β-tubulin in mosquito larvae via immersion. Produced dsRNA is processed into siRNAs, which trigger RNAi to degrade β-tubulin mRNA. Disruption of β-tubulin function impairs cytoskeletal dynamics, cell division, and vesicular transport, resulting in larval mortality and offering a novel mosquito control strategy.

## 2. Materials and Methods

### 2.1. Mosquito Strains

*Ae. albopictus* mosquitoes were collected in Guangzhou, and *Cx. quinquefasciatus* mosquitoes were collected in Haikou. Both species were continuously reared in the State Key Laboratory of Pathogen and Biosecurity under controlled conditions (26 ± 1 °C, 75 ± 5% relative humidity, and a 14:10 h light/dark photoperiod). Ventilation was performed twice daily to maintain colony health. Insecticide susceptibility was evaluated using the larval bioassay method described in GB/T 26347-2010 [26]. Based on LC50 values determined from dose–response assays with deltamethrin, the tested mosquito population exhibited approximately 700-fold resistance (resistance ratio, RR = LC50 of field population/LC50 of susceptible strain) compared with the laboratory-susceptible strain, indicating a very high level of pyrethroid resistance. Mosquitoes were blood-fed on mice three days after eclosion to induce oviposition. After a three-day blood-feeding period, the mosquitoes were transferred to oviposition cups.

### 2.2. Plasmids and Bacterial Strains

The L4440 plasmid was preserved in the State Key Laboratory of Pathogen and Biosecurity. The EGFP-L4440 plasmid used as the negative control was also obtained from a previously established laboratory stock maintained in the same laboratory. The corresponding bacterial strains were revived from glycerol stocks and cultured for subsequent dsRNA production. The bacterial strain HT115 (DE3) used in this study was obtained from Beijing Zhuangmeng International Biotechnology Co., Ltd., Beijing, China.

### 2.3. The Construction of Vectors and Bacterial Transformation Process

#### 2.3.1. Primer Design

The β-tubulin sequences of *Ae. albopictus* (GenBank accession no. XM_019696866.4), *Cx. quinquefasciatus* (XM_038251688.1), and EGFP (JQ064510.1) were retrieved from the NCBI database. Candidate primer pairs were first screened within the β-tubulin coding sequence (CDS) using the primer-assisted design function of BioXM 2.7.1. The genomic region amplified by each candidate primer pair was then defined as a potential dsRNA target fragment. These candidate fragments were subjected to BLASTn searches against the NCBI nucleotide database to identify homologous sequences from non-target organisms. The retrieved homologous sequences were subsequently aligned with the candidate target fragments using the multiple-sequence alignment function of BioXM 2.7.1 to evaluate sequence specificity and potential off-target risk. Candidate fragments containing a continuous 21 nt sequence perfectly matched to a non-target homologous sequence were excluded, and fragments meeting the above criteria were retained for dsRNA synthesis.

Primers for dsRNA synthesis and qPCR were designed using BioXM 2.7.1 and SnapGene 6.0.2 software. XhoI and SacI restriction sites were added to the 5′ ends of the primers to facilitate directional cloning into the L4440 vector. Primers were designed according to standard primer design criteria, including a primer length of 18–30 bp, a GC content of 40–60%, and a Tm difference of less than 2 °C between the forward and reverse primers. RPL8 and Actin were used as reference genes for qPCR analysis in *Cx. quinquefasciatus* and *Ae. albopictus*, respectively. The designed primers and their corresponding sequences are listed in Table 1.

#### 2.3.2. RNA Extraction and cDNA Synthesis

Total RNA was extracted from both mosquito species using RNAiso Blood (TAKARA, Beijing, China). After measuring the RNA concentration, cDNA synthesis was performed using PrimeScript RT Master Mix (TAKARA, Beijing, China). In the first-step reaction, 2.0 μL of 5× gDNA Eraser Buffer, 1.0 μL of gDNA Eraser, 1.0 μL of total RNA, and RNase-free ddH_2_O were added to a final volume of 10.0 μL. The reaction was incubated at 42 °C for 2 min, then stored at 4 °C. In the second step, 10.0 μL of the Step 1 reaction, 1.0 μL of PrimeScript RT Enzyme Mix I, 1.0 μL of RT Primer Mix, 4.0 μL of 5× PrimeScript Buffer 2, and 4.0 μL of RNase-free ddH_2_O were combined to a final volume of 20.0 μL. The reaction was carried out at 37 °C for 15 min, followed by 85 °C for 5 s, and then held at 4 °C.

#### 2.3.3. Target Fragment Amplification

The L4440-β-tubulin expression vectors were constructed by amplifying β-tubulin fragments using EasyTaq^®^ PCR SuperMix (TAKARA, Beijing, China). The PCR reaction mixture consisted of 12.5 µL of 2× EasyTaq^®^ PCR SuperMix (+dye), 1 µL of forward primer, 1 µL of reverse primer, 8.5 µL of nuclease-free water, and 2 µL of cDNA. PCR was performed with an initial denaturation at 95 °C for 5 min, followed by 35 cycles of 95 °C for 30 s, annealing at 59 °C (*Ae. albopictus*), 57 °C (*Cx. quinquefasciatus*) for 30 s, and extension at 72 °C for 1 min, with a final extension at 72 °C for 10 min. PCR products were stored at 4 °C and verified by agarose gel electrophoresis.

#### 2.3.4. Ligation and Transformation

Purified PCR products were ligated into the pEASY-T1 vector using the pEASY-T1 Simple Cloning Kit (TransGen Biotech, Beijing, China) at a 1:4 (vector:insert) ratio and incubated at 37 °C for 20 min. The ligation mixture was transformed into 50 μL of Trans-T1 competent cells (TransGen Biotech) by heat shock (42 °C, 30 s) after incubation on ice for 30 min, followed by recovery on ice for 2 min. Transformed cells were cultured in 500 μL of SOC medium at 37 °C and 200 rpm for 1 h, plated onto LB agar containing ampicillin (TransGen Biotech), and incubated overnight at 37 °C. A single colony was inoculated into LB broth supplemented with ampicillin and cultured at 37 °C for 12–16 h. Positive clones were screened by colony PCR using M13-47 and RV-W universal primers and confirmed by Sanger sequencing.

#### 2.3.5. Construction of L4440 β-Tubulin Recombinant Bacteria

The recombinant plasmid and L4440 vector were confirmed by Sanger sequencing and digested with SacI and XhoI (New England Biolabs, Ipswich, MA, USA). Each 100 μL digestion reaction contained 5 μg DNA, 5 μL 10× rCutSmart buffer, 1 μL of each restriction enzyme, and nuclease-free water, followed by incubation at 37 °C for 1 h. The digested fragments were purified by agarose gel electrophoresis and ligation was carried out using T4 DNA ligase (New England Biolabs) for 16 h at 25 °C, with the following components: 1 μL of T4 ligase, 2 μL of buffer, 5 μL of the linearized vector, and 2 μL of the target fragment, bringing the total volume to 10 μL.

The ligation products were transformed into *E. coli* HT115 (DE3) competent cells (TransGen Biotech) and plated onto LB agar containing ampicillin and tetracycline (50 μg/mL, TransGen Biotech). After incubation at 37 °C for 12–16 h, positive colonies were cultured in LB broth supplemented with ampicillin and tetracycline for 12–16 h at 37 °C with shaking. Recombinant plasmids were verified by colony PCR and Sanger sequencing.

### 2.4. Induction of β-Tubulin dsRNA Expression

The recombinant HT115 strain was inoculated into LB medium containing Amp+ and Tet+, and cultured overnight at 37 °C with shaking at 200 rpm. A 1:10 dilution of the overnight culture was then inoculated into fresh LB medium with Amp+, and cultured at 37 °C with shaking at 220 rpm for 12–16 h. When the OD600 reached 0.4–0.6, IPTG (TransGen Biotech) was added to a final concentration of 1 mmol/L, and the culture was further incubated for 4 h with shaking.

Induced cultures were collected by centrifugation at 4 °C and 8000 rpm for 5 min. Total RNA was extracted from induced *E. coli* cultures using RNAiso Blood. To confirm the presence and integrity of dsRNA, dsRNA samples were treated with DNase I (TAKARA) to remove residual free DNA and RNase A (TAKARA) to digest free single-stranded RNA, while dsRNA molecules were expected to remain intact for subsequent agarose gel electrophoresis analysis. For DNase I digestion, 1.0 μL of DNase I (1 U/μL), 1.0 μL of dsRNA sample, and 6.0 μL of ddH_2_O were incubated at 37 °C for 30 min. For RNase A digestion, 0.75 μL of RNase A (1.0 μg/mL), 3.5 μL of dsRNA sample, and 5.75 μL of 1 mmol/L NaCl were incubated at 37 °C for 1 h. After DNase I and RNase A digestion, samples were treated with proteinase K (150 μg/mL final concentration) containing 0.5% SDS at 50 °C for 30 min to degrade residual nucleases. The dsRNA was subsequently purified using a magnetic-bead-based dsRNA purification kit (BelongBio, Shanghai, China), followed by elution in nuclease-free water. Because a single dsRNA preparation was insufficient for all bioassays, dsRNA was prepared in multiple batches. All dsRNA preparations were pooled into a single stock solution before concentration determination. The pooled dsRNA stock was diluted to the required concentrations and used for larval immersion assays.

### 2.5. Larval Survival Bioassay of dsβ-Tub dsRNA on Mosquitoes

Three concentrations of β-tubulin dsRNA (500, 750, and 1000 ng/μL) were tested using 1-day-old larvae (12–24 h after hatching). For each concentration, three parallel wells containing approximately 100 larvae in 5 mL of treatment solution were prepared. The assay was independently repeated three times using larvae from different egg batches; parallel wells were considered technical replicates and independent experiments biological replicates. DNase I/RNase A-treated dsRNA was diluted with sterile enzyme-free water to the required concentrations, while dsEGFP dsRNA and sterile enzyme-free water served as negative controls. During exposure, larvae were provided with a small amount of fish food. Surviving larvae were collected at 24, 48, and 72 h for RT-qPCR analysis and were excluded from survival calculations. At each time point, five larvae were randomly selected from each treatment group in each independent experiment, yielding 15 larvae per group in total. RNA was extracted individually from each larva, and RT-qPCR was performed in technical triplicate.

RT-qPCR was performed using the One Step SYBR Green RT-qPCR Kit (Vazyme Biotech, Nanjing, China). Each 20 μL reaction contained 10 μL of 2× One Step SYBR Green Mix, 0.4 μL of 50× ROX Reference Dye 1, 1 μL of One Step Green Enzyme Mix, 4 μL of forward primer, 4 μL of reverse primer, 3 μL of RNA template, and nuclease-free water to a final volume of 20 μL.

The amplification program consisted of reverse transcription at 50 °C for 3 min, initial denaturation at 95 °C for 30 s, followed by 40 cycles of 95 °C for 10 s and 60 °C for 30 s. Melting curve analysis was performed at 95 °C for 15 s, 60 °C for 60 s, and 95 °C for 15 s. Relative β-tubulin expression levels were calculated using the 2^−ΔΔCt^ method with the corresponding reference gene. Statistical analyses were performed using two-way ANOVA followed by Dunnett’s multiple comparisons test in GraphPad Prism.

### 2.6. Transcriptomic and Proteomic Analysis of Mosquitoes After β-Tub dsRNA Interference

#### 2.6.1. Transcriptome Sequencing and Bioinformatic Analysis

For transcriptomic analysis, approximately 15–30 surviving larvae were collected 24 h after β-tubulin dsRNA treatment, pooled as one biological sample, immediately frozen in liquid nitrogen, and stored at −80 °C until RNA extraction. Total RNA quality was assessed by agarose gel electrophoresis, NanoDrop, Qubit, and capillary electrophoresis. For each sample, 1 μg of total RNA was used for library construction. Poly(A)+ mRNA was enriched with Oligo(dT) magnetic beads, fragmented, and reverse-transcribed using random primers. After second-strand synthesis, cDNA was end-repaired, dA-tailed, adaptor-ligated, size-selected with DNA Clean Beads, and amplified by PCR. Indexed libraries were pooled and sequenced on an Illumina platform using a 2 × 150 bp paired-end strategy.

Raw FASTQ reads were filtered using fastp v0.24.1 to remove adaptor sequences and low-quality reads, with the following criteria: Phred quality cutoff of 20, maximum error rate of 10%, minimum read length of 75 bp, and no more than five ambiguous bases. Read quality was assessed using Fast QC v0.10.1. Clean reads were mapped to the corresponding *Ae. albopictus* and *Cx. quinquefasciatus* reference genomes using HISAT2 v2.2.1. Gene-level counts were generated with HTSeq v0.6.1, excluding multi-mapped reads and reads overlapping multiple genes. Differentially expressed genes were identified using DESeq2 v1.26.0, with |log_2_FC| ≥ 1 and FDR (false discovery rate) < 0.05 as thresholds. GO (Gene Ontology) enrichment was performed using GOSeq v1.34.1 with gene-length bias correction and visualized using topGO v2.18.0. KEGG (Kyoto Encyclopedia of Genes and Genomes); pathway enrichment was tested by hypergeometric analysis. GO terms and KEGG pathways with FDR or q-value < 0.05 were considered significantly enriched. PCA was performed in R (version 2.0) using the prcomp function based on FPKM values.

#### 2.6.2. Proteomic Profiling and Bioinformatic Analysis

For proteomic analysis, approximately 15–30 surviving larvae were pooled per biological sample and stored at −80 °C. Samples were briefly thawed at 4 °C to remove residual rearing water, then immediately ground in liquid nitrogen and lysed with four volumes of buffer containing 1% SDS and 1% protease inhibitor. Lysates were heated at 95 °C for 10 min, sonicated on ice for 3 min, and centrifuged at 12,000× *g* for 5 min at 4 °C. The supernatants were collected for BCA protein quantification, and protein quality was assessed by 12% SDS-PAGE followed by Coomassie Brilliant Blue staining.

Proteins were reduced with 100 mM dithiothreitol at 95 °C for 5 min and alkylated with 100 mM iodoacetamide in the dark for 30 min. After washing with 8 M urea buffer and 25 mM ammonium bicarbonate, proteins were digested with trypsin at 37 °C for 20 h, and digestion was stopped with 10% formic acid. Peptides were desalted using C18 tips, eluted with 70% acetonitrile containing 0.1% formic acid, and dried under vacuum.

Peptides were separated on a Vanquish Neo UHPLC (ultra-high-performance liquid chromatography) system with an EASY-Spray C18 column (2 μm, 150 μm × 15 cm; Thermo Fisher Scientific, Waltham, MA, USA) and analyzed using an Orbitrap Astral mass spectrometer (Thermo Fisher Scientific) in data-independent acquisition mode. The MS1 scan range was 380–980 *m*/*z* at a resolution of 240,000, with a normalized AGC (automatic gain control) target of 500% and a maximum injection time of 5 ms. MS2 spectra were acquired with 300 DIA windows, a 2 *m*/*z* isolation window, 25 eV HCD (higher-energy collisional dissociation) collision energy, a normalized AGC target of 500%, and a maximum injection time of 3 ms.

DIA-MS data were processed using DIA-NN v1.9.2 and searched against the UniProt protein databases for *Aedes albopictus* (release 2026_02; 34,727 entries) and *Culex quinquefasciatus* (release 2026_02; 20,064 entries). Trypsin was specified as the digestion enzyme, allowing one missed cleavage. Carbamidomethylation of cysteine was set as a fixed modification, whereas methionine oxidation and protein N-terminal acetylation were set as variable modifications. Protein identifications were accepted at 99% confidence with an FDR ≤ 1%.

### 2.7. Bioinformatic Off-Target Analysis

To assess the potential off-target effects on non-target organisms, the target sequence of *Aedes albopictus* was compared with homologous sequences from four representative non-target species, *Danio rerio*, *Daphnia magna*, *Apis mellifera*, and *Procambarus clarkii*. The corresponding homologous sequences were retrieved from the NCBI database using the accession numbers NM_131327.1, GDIP01026603.1, XM_396338.6, and XM_069308548.1, respectively. The sequences were downloaded and subjected to multiple sequence alignment using BioXM software (version 2.7.1). Sequence similarity between the *Ae. albopictus* target sequence and the homologous sequences of the four non-target species was then evaluated to assess the potential for off-target effects.

### 2.8. Statistics

Statistical analyses of survival, mortality, and RT-qPCR data were performed using GraphPad Prism 8.0. Pairwise comparisons between two groups were conducted using Student’s *t*-test, whereas comparisons among multiple groups were performed using one-way analysis of variance (ANOVA). Data are presented as means ± standard deviations (SDs), and *p* < 0.05 was considered statistically significant.

For transcriptomic analysis, differentially expressed genes were identified using DESeq2 based on normalized read counts. Genes with |log_2_ fold change| ≥ 1 and FDR < 0.05 were considered significantly differentially expressed. GO enrichment analysis was performed using GOSeq, and KEGG pathway enrichment was assessed using a hypergeometric test. GO terms and KEGG pathways with FDR or q-value < 0.05 were considered significantly enriched.

For proteomic analysis, protein identification was performed using DIA-NN, and the false discovery rate (FDR) was controlled at ≤1%. Differentially expressed proteins were screened according to the fold-change and statistical significance criteria used in the proteomic data analysis. For differential protein abundance analysis, comparisons between two groups were performed using Student’s *t*-test, whereas comparisons involving multiple groups were performed using one-way analysis of variance (ANOVA) followed by Tukey’s multiple-comparison test.

## 3. Result

### 3.1. Engineered E. coli Successfully Produced Two Mosquito dsRNAs

**Figure 1:**
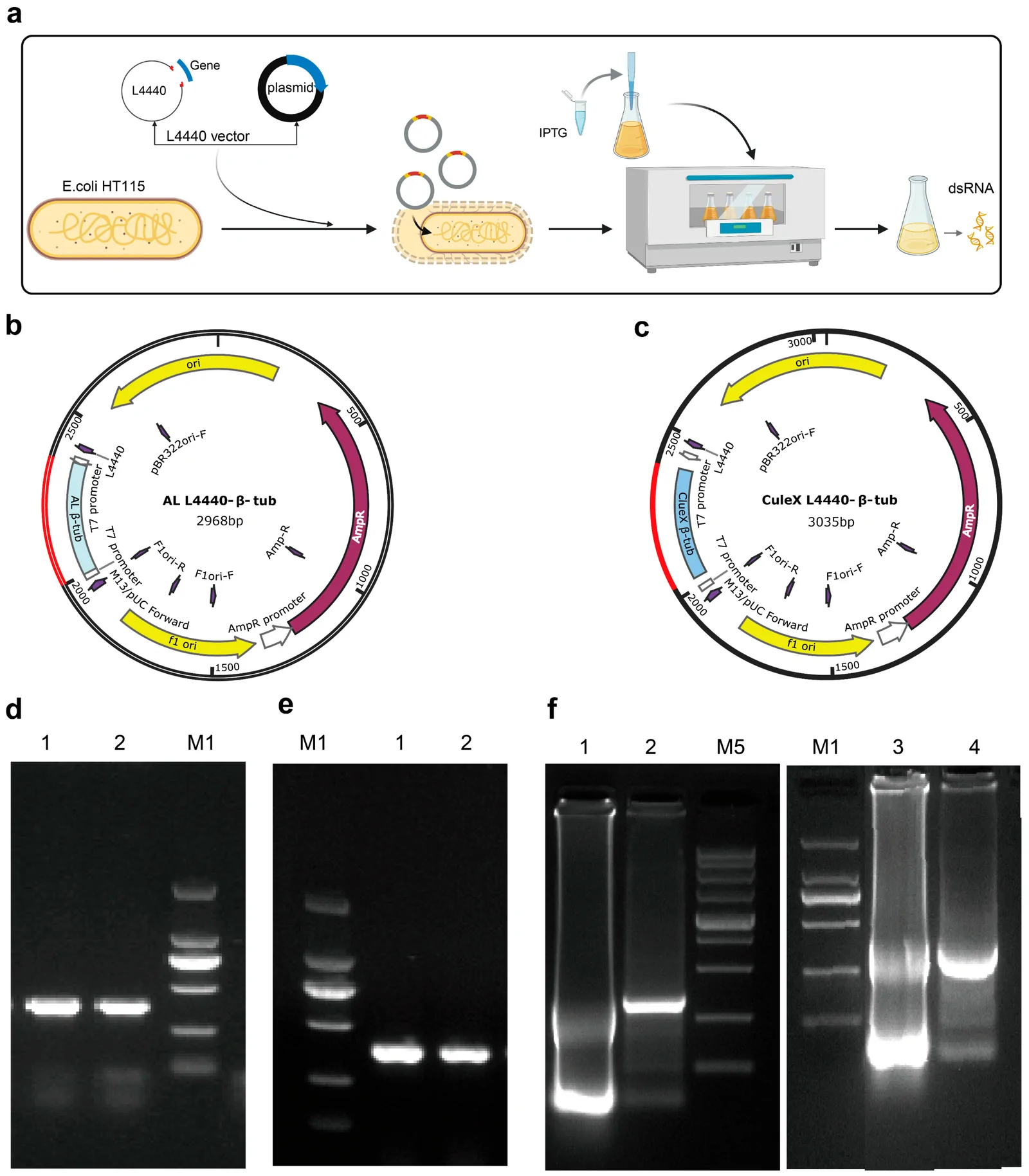
Construction and induced expression of synthetic dsRNA expression vectors in engineered bacteria. (**a**) Schematic representation of dsRNA synthesis in *E. coli* HT115 (DE3). (**b**) Schematic representation of the double-enzyme digestion and ligation of the *Ae. albopictus* β-tubulin gene. (**c**) Schematic representation of the double-enzyme digestion and ligation of the *Cx. quinquefasciatus* β-tubulin gene. (**d**) Electrophoresis of target fragments (lane 1: *Cx. quinquefasciatus*; lane 2: *Ae. albopictus*). (**e**) Electrophoresis of recombinant bacterial cultures (lane 1: *Cx. quinquefasciatus*; lane 2: *Ae. albopictus*). (**f**) Electrophoresis of dsRNA after IPTG induction. Lanes 1 and 3 show dsRNA samples from *Ae. albopictus* and *Cx. pipiens pallens*, respectively, before DNase I and RNase A digestion, whereas lanes 2 and 4 show the corresponding samples after digestion. M1, 2000 bp DNA marker (2000, 1000, 750, 500, 250, and 100 bp); M5, 5000 bp DNA marker (5000, 3000, 2000, 1500, 1000, 750, 500, 250, and 100 bp).

The dsRNA biosynthesis process was constructed using the HT115-L4440 system, as illustrated in Figure 1a. The β-tubulin genes of *Ae. albopictus* and *Cx. quinquefasciatus* were selected as RNAi targets, and specific primers were designed to include XhoI and SacI restriction sites. Homologous recombination was used to construct the L4440-β-tubulin recombinant expression vectors for both mosquito species (Figure 1b,c). PCR amplification confirmed target fragment sizes of 363 bp for *Ae. albopictus* and 361 bp for *Cx. quinquefasciatus* (Figure 1d). These fragments were then ligated into the double-digested L4440 vector using T4 DNA ligase and transformed into *E. coli* HT115 (DE3). Positive recombinant strains were identified by colony PCR (Figure 1e), confirming the successful construction of the β-tubulin L4440 expression vectors. After IPTG (isopropyl β-D-1-thiogalactopyranoside) induction at a final concentration of 1 mmol/L, the culture was centrifuged, and total RNA was extracted. Treatment with DNase I and RNase A revealed a prominent 360 bp target band (Figure 1f), confirming successful induction of dsβ-tubulin and efficient dsRNA production. Supplementary Figure S1 shows comparative agarose gel electrophoresis profiles of β-tubulin dsRNA products from *Ae. albopictus* and *Cx. quinquefasciatus*, as well as dsEGFP, before and after DNase I/RNase A treatment. In addition, the target sequences were compared with homologous sequences from representative non-target organisms, including zebrafish (*Danio rerio*), *Daphnia magna*, honeybee (*Apis mellifera*), and red swamp crayfish (*Procambarus clarkii*). The alignment results are provided in Supplementary Sequence Alignments S1 and S2. No continuous 21 nt perfect matches were identified in the examined sequences, providing a preliminary bioinformatic assessment of sequence-specific off-target risk.

### 3.2. β-Tubulin Silencing Induces Conserved Larvicidal Activity in Susceptible and Resistant Ae. albopictus and Cx. quinquefasciatus

**Figure 2:**
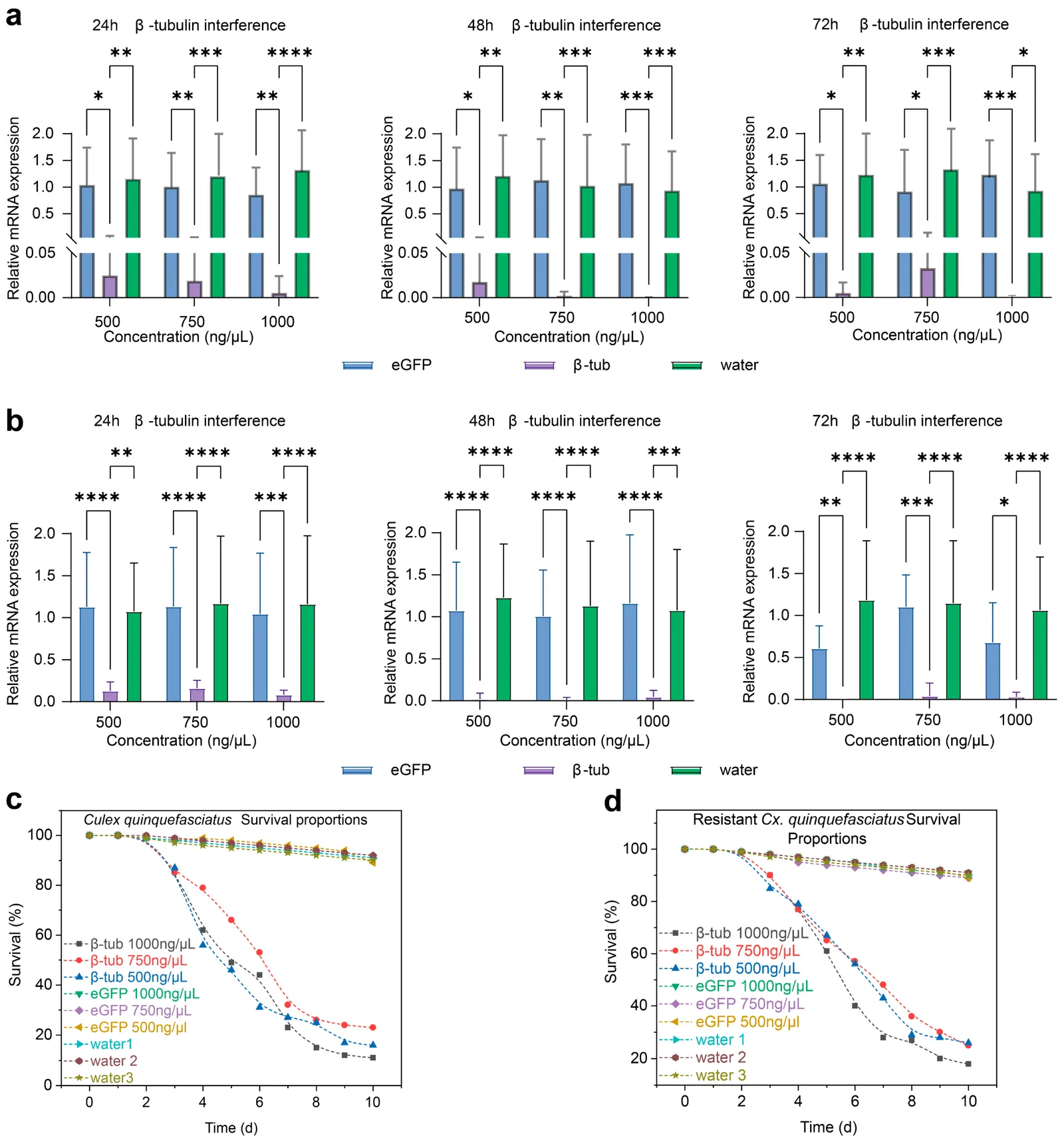
Disruption Effects of β-tubulin dsRNA on *Cx. quinquefasciatus* Larvae. (**a**,**b**) RT-qPCR analysis of β-tubulin expression in susceptible and resistant larvae at 24–72 h after β-tubulin dsRNA treatment. Data are presented as means ± SD from 15 individual larvae sampled across three independent biological replicates (five larvae per biological replicate). **** *p* < 0.001, *** *p* < 0.005, ** *p* < 0.01, and * *p* < 0.05. (**c**,**d**) Larvicidal effects of β-tubulin dsRNA on susceptible and resistant *Cx. quinquefasciatus* larvae. Data are presented as means from three biological replicates, with 100 larvae per replicate.

RT-qPCR analysis showed that β-tubulin dsRNA significantly suppressed β-tubulin expression at all tested concentrations in susceptible and resistant *Cx. quinquefasciatus* larvae, with maximum reductions of 571-fold and 333-fold, respectively (Figure 2a,b). Consistent with the silencing effect, β-tubulin dsRNA markedly increased larval mortality, reaching 85.00% in the susceptible strain and 89.00% in the resistant strain, compared with 8.00% and 10.00% in the corresponding controls, respectively (Figure 2c,d). These results demonstrate that β-tubulin dsRNA efficiently silences the target gene and induces substantial larval mortality in both strains.

**Figure 3:**
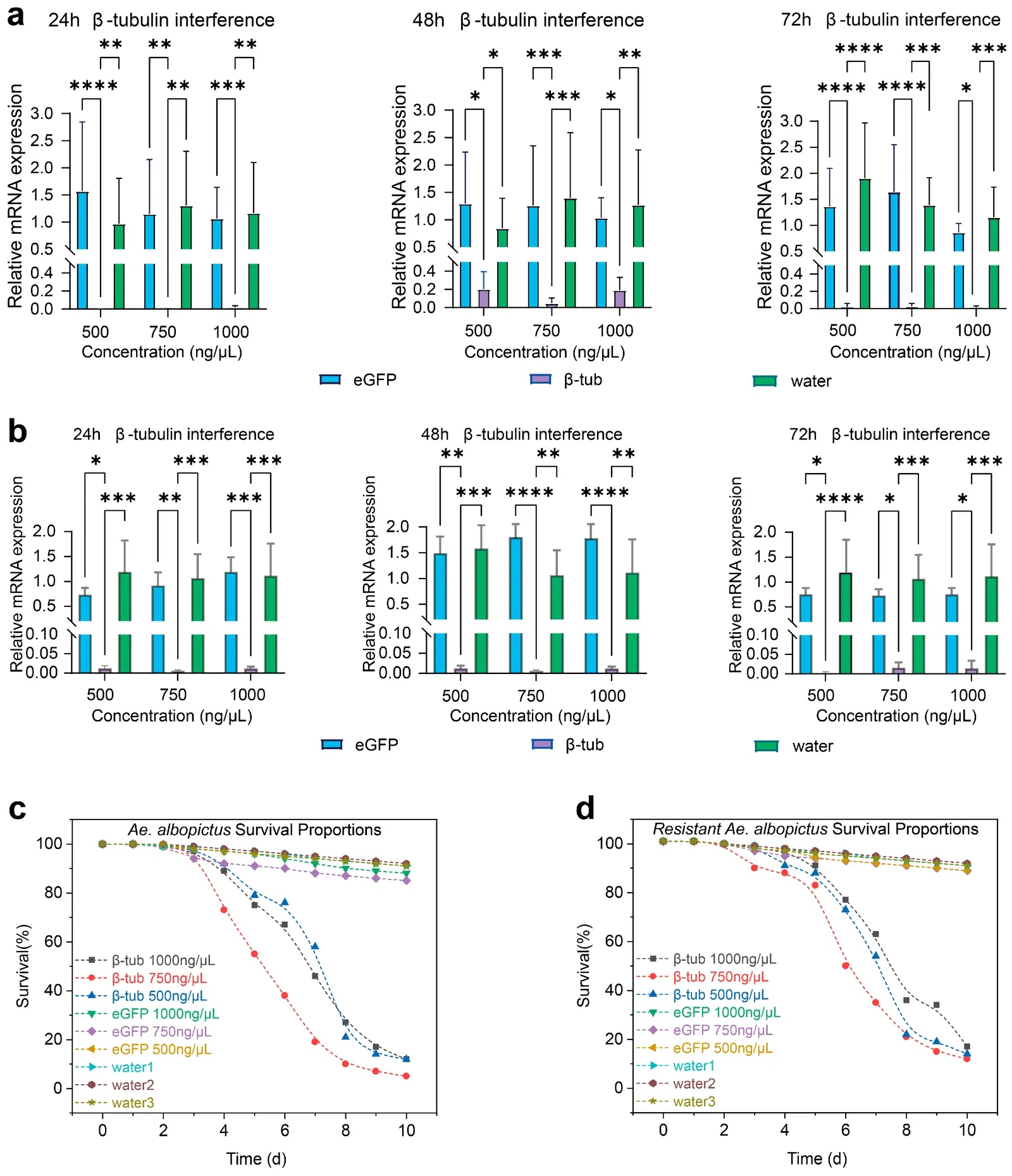
Disruption Effects of β-tubulin dsRNA on *Ae. albopictus* Larvae. (**a**,**b**) RT-qPCR analysis of β-tubulin expression in susceptible and resistant larvae at 24–72 h after β-tubulin dsRNA treatment. Data are presented as means ± SD from 15 individual larvae sampled across three independent biological replicates (five larvae per biological replicate). **** *p* < 0.001, *** *p* < 0.005, ** *p* < 0.01, and * *p* < 0.05. (**c**,**d**) Larvicidal effects of β-tubulin dsRNA on susceptible and resistant *Ae. albopictus* larvae. Data are presented as means from three biological replicates, with 100 larvae per replicate.

Similarly, β-tubulin dsRNA significantly suppressed β-tubulin expression at all tested concentrations in susceptible and resistant *Ae. albopictus* larvae, with maximum reductions of 157-fold and 337-fold, respectively (Figure 3a,b). This silencing effect was accompanied by marked larval mortality, which reached 91.00% in the susceptible strain and 82.00% in the resistant strain, compared with 9.00% and 8.00% in the corresponding controls (Figure 3c,d). The comparable responses observed in susceptible and resistant larvae further indicate that β-tubulin-targeted RNAi remains effective across different insecticide susceptibility backgrounds.

### 3.3. β-Tubulin dsRNA Induced Species-Specific Transcriptomic Responses in Ae. albopictus and Cx. quinquefasciatus

**Figure 4:**
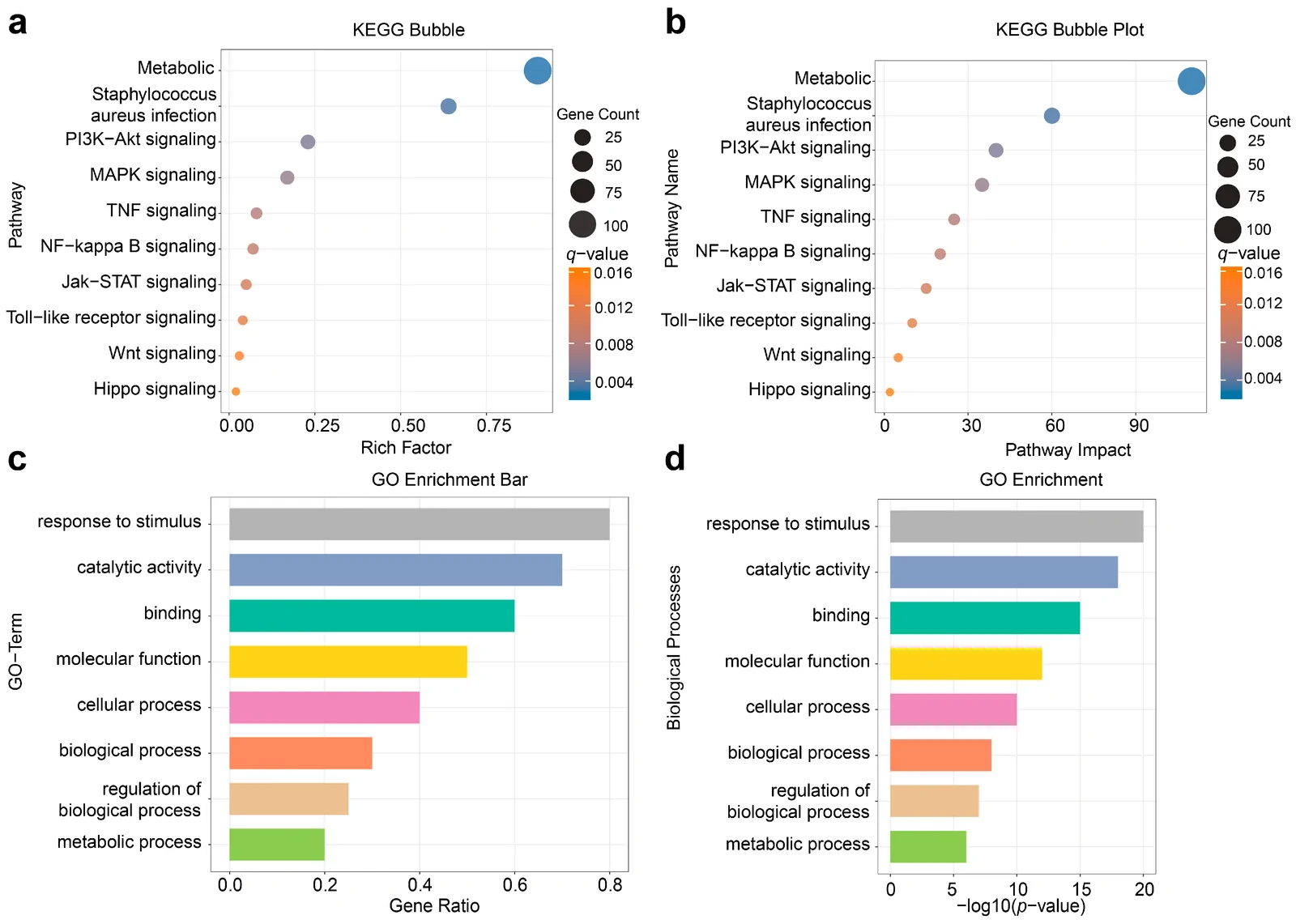
Transcriptomic analysis of *Cx. quinquefasciatus* mosquitoes following β-tubulin dsRNA interference. (**a**) KEGG pathway enrichment analysis of differentially expressed genes (DEGs). The *x*-axis represents the Rich Factor, defined as the proportion of DEGs among all genes annotated to a given pathway. Bubble size represents the number of DEGs enriched in each pathway (Gene Count), and bubble color represents the q-value, with lower q-values indicating higher statistical significance. (**b**) KEGG pathway topology analysis. The *x*-axis represents the Pathway Impact value, a topology-based parameter reflecting the relative influence of affected genes within each pathway. Bubble size indicates Gene Count, and color represents the q-value. (**c**) Gene Ontology (GO) enrichment analysis. The *x*-axis represents the Gene Ratio, defined as the proportion of DEGs assigned to each GO category. (**d**) Significance of GO enrichment. The *x*-axis represents −log10(*p* value), with larger values indicating greater statistical significance of enrichment.

Transcriptomic profiling was performed to characterize gene expression changes following β-tubulin dsRNA treatment in *Cx. quinquefasciatus*, and the RNA-seq data were deposited in the NCBI Sequence Read Archive under BioProject accession number PRJNA1504848. KEGG enrichment analysis showed that differentially expressed genes (DEGs) were mainly enriched in metabolic pathways and several conserved signaling pathways, including PI3K–Akt, MAPK, TNF, NF-κB, Jak–STAT, Toll-like receptor, Wnt, and Hippo signaling (Figure 4a). Several pathways also showed relatively high impact values (Figure 4b). GO enrichment analysis identified significant enrichment of DEGs associated with response to stimulus, catalytic activity, binding, regulation of biological processes, and metabolic processes (Figure 4c,d).

**Figure 5:**
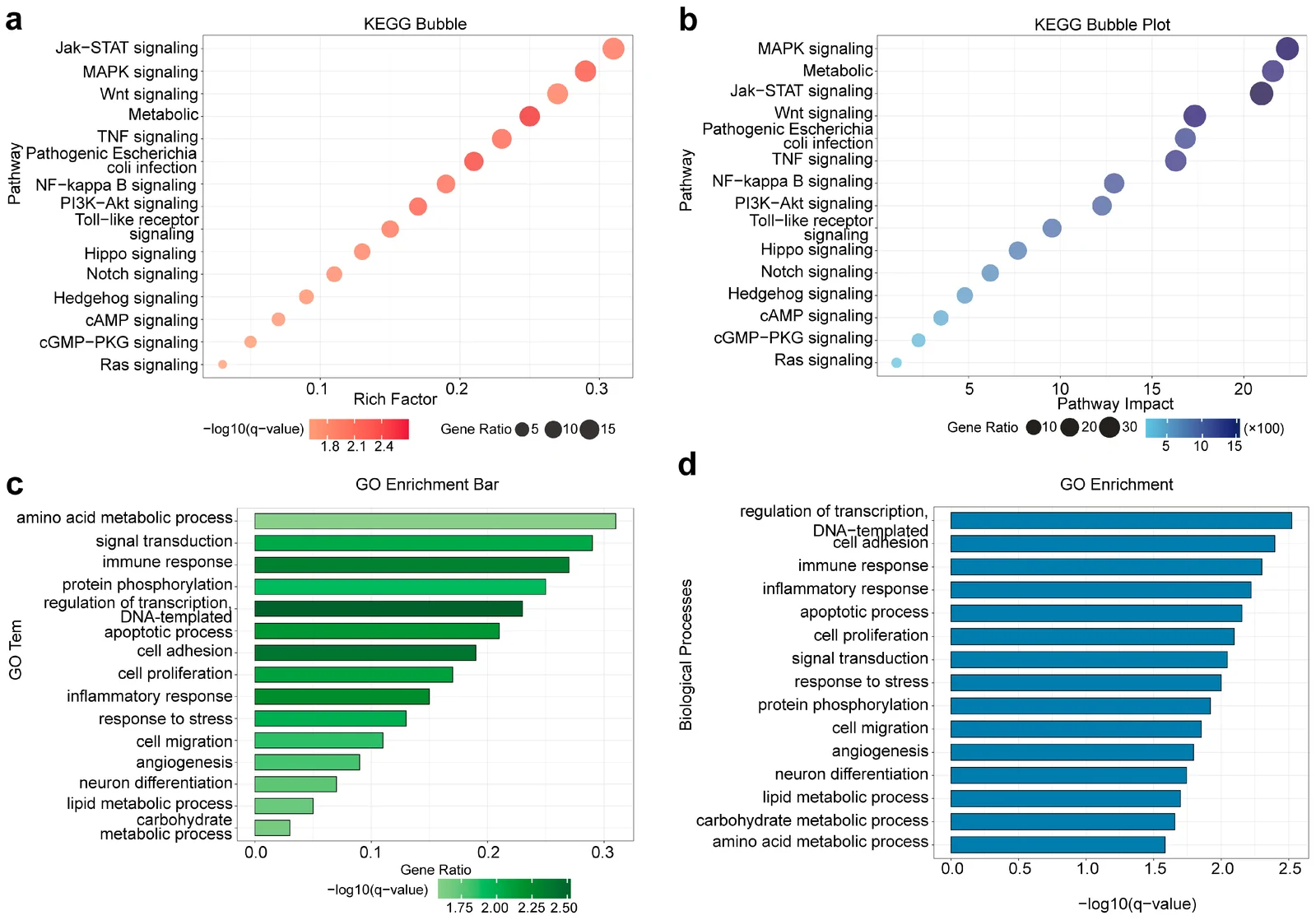
Transcriptomic profiling of *Ae. albopictus* following β-tubulin dsRNA interference. (**a**) KEGG pathway enrichment analysis of differentially expressed genes (DEGs). The *x*-axis represents the Rich Factor, defined as the proportion of DEGs among all genes annotated to a given pathway. Bubble size represents the Gene Ratio, and bubble color represents −log10(q-value), with larger values indicating greater statistical significance. (**b**) KEGG pathway topology analysis. The *x*-axis represents the Pathway Impact value, a topology-based parameter reflecting the relative influence of affected genes within each pathway. Bubble size represents the Gene Ratio. (**c**) Gene Ontology (GO) enrichment analysis of DEGs. The *x*-axis represents the Gene Ratio, defined as the proportion of DEGs assigned to each GO term, and bar color corresponds to −log10(q-value). (**d**) Significance of GO enrichment. The *x*-axis represents −log10(q-value), with larger values indicating greater statistical significance of enrichment.

A broadly similar transcriptional response was observed in *Ae. albopictus* following β-tubulin dsRNA treatment. KEGG enrichment analysis showed that differentially expressed genes (DEGs) were mainly enriched in multiple conserved signaling pathways, including MAPK, Jak–STAT, Wnt, PI3K–Akt, NF-κB, TNF, and Toll-like receptor signaling, as well as metabolic pathways (Figure 5a). Several pathways also showed relatively high impact values (Figure 5b). GO enrichment analysis identified significant enrichment of genes associated with signal transduction, immune response, protein phosphorylation, transcriptional regulation, apoptosis, cell adhesion, cell proliferation, and metabolic processes (Figure 5c,d).

Comparison of the enrichment profiles between *Cx. quinquefasciatus* and *Ae. albopictus* revealed substantial overlap in metabolic and conserved signaling pathways, including PI3K–Akt, MAPK, TNF, NF-κB, Jak–STAT, Toll-like receptor, and Wnt signaling. These shared responses suggest a conserved molecular response to β-tubulin silencing across mosquito species. Notably, *Ae. albopictus* exhibited broader enrichment of pathways associated with development, cell fate determination, and signal transduction, indicating species-specific differences in the downstream consequences of β-tubulin knockdown.

### 3.4. β-Tubulin dsRNA Induced Distinct Proteomic Responses in Ae. albopictus and Cx. quinquefasciatus Larvae

**Figure 6:**
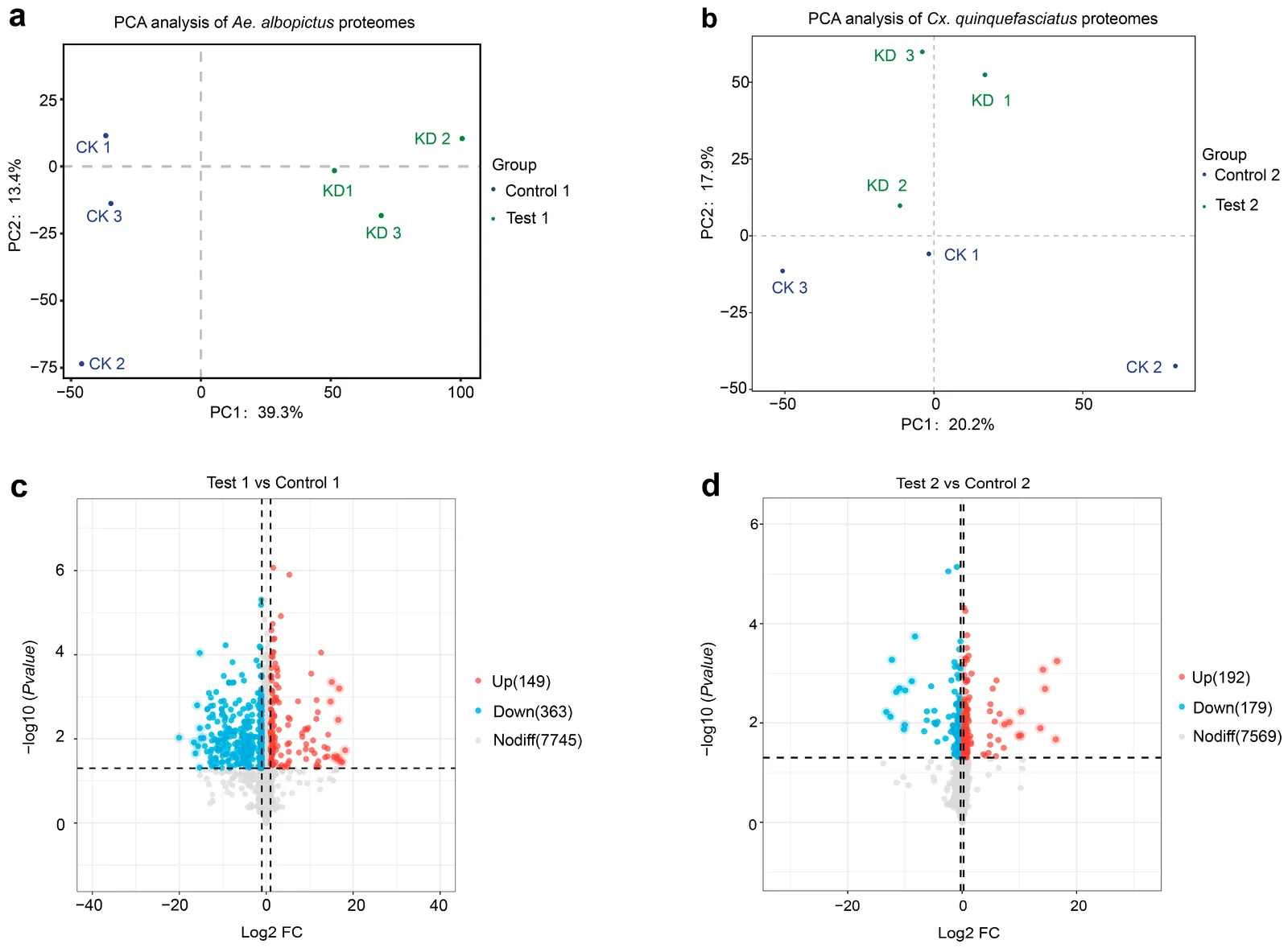
Proteomic changes following β-tubulin knockdown in two mosquito species. (**a**,**b**). Principal component analysis (PCA) of proteomic profiles of *Aedes albopictus* (**a**) and *Culex quinquefasciatus* (**b**) following β-tubulin knockdown. Each point represents a biological replicate. The x- and y-axes indicate the first (PC1) and second (PC2) principal components, respectively, with the percentage of variance explained by each component shown. (**c**,**d**) Volcano plots of differentially expressed proteins (DEPs) in *Ae. albopictus* (**c**) and *Cx. quinquefasciatus.* (**d**) The *x*-axis shows the log2 fold change (log2FC) between the knockdown and control groups, and the *y*-axis shows −log10(*p* value). Significantly upregulated and downregulated proteins are shown in red and blue, respectively, and non-significant proteins in grey. Numbers in parentheses indicate the number of proteins in each category. CK, control group; KD, β-tubulin knockdown group; PCA, principal component analysis; PC1, first principal component; PC2, second principal component; DEP, differentially expressed protein. Vertical dashed lines correspond to log_2_ fold change = ±1, and horizontal dashed line represents *p* value = 0.05. Note that the x axis scale differs between panel c and d, leading to visual differences in the apparent position of vertical thresholds.

Proteomic profiling revealed distinct responses to β-tubulin dsRNA treatment in *Ae. albopictus* and *Cx. quinquefasciatus* larvae. The proteomics data have been deposited in the iProX database under ProteomeXchange accession number PXD082041. In *Ae. albopictus*, principal component analysis (PCA) showed clear separation between the β-tubulin knockdown and control samples, with PC1 and PC2 explaining 39.3% and 13.4% of the total variance, respectively (Figure 6a). Differential expression analysis identified 149 upregulated and 363 downregulated proteins following β-tubulin knockdown (Figure 6c).

Similarly, the proteomic profiles of *Cx. quinquefasciatus* larvae showed separation between the knockdown and control groups, with PC1 and PC2 accounting for 20.2% and 17.9% of the total variance, respectively (Figure 6b). A total of 192 proteins were upregulated and 179 proteins were downregulated following β-tubulin knockdown (Figure 6d).

The two mosquito species showed different patterns of proteomic response to β-tubulin knockdown. In *Ae. albopictus*, downregulated proteins outnumbered upregulated proteins, whereas *Cx. quinquefasciatus* showed a more balanced distribution of up- and downregulated proteins. Functional enrichment analyses further revealed differences in the enriched pathways and biological processes between the two species (Supplementary Figure S2).

Overall, these results indicate that β-tubulin knockdown is associated with distinct proteomic response patterns in *Ae. albopictus* and *Cx. quinquefasciatus* larvae.

## 4. Discussion

The increasing prevalence of insecticide resistance in mosquito populations has created an urgent demand for alternative vector-control strategies that are both effective and environmentally sustainable [2,3,4,5]. In this study, we utilized the established *E. coli* HT115-L4440 dsRNA expression system to produce β-tubulin-targeting dsRNA and demonstrated that β-tubulin RNA interference effectively reduced larval survival in both *Ae. albopictus* and *Cx. quinquefasciatus*. These findings support β-tubulin as a promising RNAi target for mosquito control because of its conserved and essential biological functions. The results are consistent with previous reports in *Aedes aegypti* [25] and provide further experimental evidence that β-tubulin is a conserved and vulnerable molecular target for RNAi-based mosquito control.

A major finding of this study is that β-tubulin dsRNA induced strong gene silencing and high larval mortality in two epidemiologically important mosquito species. RT-qPCR analysis confirmed marked suppression of β-tubulin expression after dsRNA treatment, while larval bioassays showed substantial reductions in survival, particularly at higher dsRNA concentrations. These effects are consistent with the essential role of β-tubulin in maintaining microtubule structure, intracellular transport, mitotic spindle formation, and cellular homeostasis. Disruption of these processes likely compromises tissue development, nutrient absorption, and larval growth, ultimately leading to developmental arrest and mortality [27,28].

Nevertheless, previous studies targeting β-tubulin have reported variable RNAi outcomes among different insect species and experimental systems. Although β-tubulin is a conserved and essential cellular component, the efficiency of β-tubulin silencing and the resulting phenotypic effects are not necessarily uniform across studies [29]. Such variation may reflect differences in mosquito species, developmental stages, target sequences, dsRNA delivery approaches, doses, and experimental conditions. In particular, microinjection can provide direct and efficient dsRNA delivery but has limited practical value for large-scale mosquito control, whereas soaking- or immersion-based exposure depends more strongly on larval uptake capacity and dsRNA stability in the surrounding aquatic environment [30]. Differences in dsRNA production methods, including chemically or enzymatically synthesized dsRNA and bacterially produced dsRNA, may also affect dsRNA quality, stability, and biological activity [31]. Therefore, comparisons among RNAi studies should consider not only the target gene but also the technical framework used for dsRNA preparation, delivery, and evaluation.

The design of the dsRNA itself may further contribute to variation in RNAi efficiency. Previous studies targeting β-tubulin have used different transcript regions and dsRNA lengths, which may influence cellular uptake, processing, and subsequent gene silencing. Longer dsRNA fragments may generate a broader population of siRNAs, whereas shorter fragments may provide greater sequence specificity [32]. Sequence composition, including GC content, may also influence dsRNA stability and accessibility [33]. In the present study, 362 bp and 365 bp β-tubulin dsRNA fragments were used for *Cx. quinquefasciatus* and *Ae. albopictus*, respectively, with GC contents of 59.12% and 56.44%. These fragment lengths are within the range of relatively short β-tubulin dsRNAs used in previous mosquito RNAi studies [25]. However, because previous studies differ simultaneously in mosquito species, developmental stage, delivery method, dose, target position, and dsRNA production system, the contribution of any individual dsRNA design parameter remains difficult to isolate. Standardized comparative studies will therefore be required to determine how sequence position, fragment length, and composition influence β-tubulin RNAi efficiency across mosquito species.

Notably, comparable gene-silencing and larvicidal effects were observed in pyrethroid-susceptible and -resistant strains. This finding suggests that susceptibility to β-tubulin RNAi is not substantially diminished by the resistance mechanisms present in the tested populations, which may include pyrethroid target-site insensitivity and enhanced metabolic detoxification [34]. Because β-tubulin dsRNA acts through sequence-specific suppression of an essential cytoskeletal gene rather than through the molecular targets of conventional insecticides, it may retain activity in mosquito populations with reduced pyrethroid susceptibility. The observed changes in detoxification-associated proteins in *Cx. quinquefasciatus* are more plausibly interpreted as secondary physiological responses to β-tubulin disruption than as evidence that established detoxification mechanisms protect larvae from RNAi-mediated effects [35,36]. These findings support the potential use of RNAi targeting essential genes as a complementary strategy for managing insecticide-resistant mosquito populations. Its principal advantage lies in the ability to select and redesign target sequences independently of conventional insecticide modes of action. However, because β-tubulin is highly conserved, the proposed target specificity and potential reduction in non-target effects will require careful sequence screening and empirical assessment in non-target organisms [37].

Transcriptomic analysis revealed broadly similar responses to β-tubulin silencing in *Ae. albopictus* and *Cx. quinquefasciatus*, suggesting that disruption of this essential cytoskeletal component induces a shared physiological response in the two mosquito species. This is consistent with the fundamental roles of tubulin-based microtubules in cell division and intracellular transport [23]. In contrast, the proteomic responses were more species-specific, with *Ae. albopictus* and *Cx. quinquefasciatus* showing distinct patterns of protein-level regulation. Because β-tubulin silencing efficiency and larvicidal activity were comparable between the two species, these differences may reflect species-specific downstream or compensatory responses rather than major differences in RNAi susceptibility. The incomplete concordance between transcriptomic and proteomic responses is also consistent with the multiple regulatory processes that separate mRNA abundance from protein abundance [38]. Overall, β-tubulin silencing appears to produce a broadly conserved physiological effect while eliciting distinct molecular adaptations in the two mosquito species.

Another advantage of this study is the use of the established HT115-L4440 bacterial expression system for dsRNA production. Compared with in vitro transcription-based approaches that require enzyme-dependent synthesis and additional purification steps, bacterial expression provides a potentially scalable and cost-effective strategy for dsRNA generation [21]. In this study, dsRNA was enriched from bacterial cultures using Trizol-based total nucleic acid extraction followed by RNase A and DNase I digestion, representing a relatively simple purification workflow that has been widely adopted in microbial dsRNA production studies [8]. Under our experimental conditions, preliminary estimation based on consumable costs and dsRNA yield suggested that bacterial production may reduce the production cost per unit amount of dsRNA compared with commercial in vitro transcription. However, this estimation should be interpreted cautiously, as large-scale economic feasibility will depend on culture efficiency, dsRNA recovery, purification requirements, labor, equipment investment, and final product specifications. Further techno-economic analyses and process optimization will be required before industrial application.

Bacteria-derived dsRNA also provides a potential basis for immersion- or feeding-based delivery to mosquito larvae, which may be more compatible with practical mosquito control than direct administration by microinjection [39]. Nevertheless, several challenges must be addressed before field deployment. The crude dsRNA preparation used in this study may contain residual nucleic acid fragments and bacterial-derived components, and their potential effects on dsRNA stability, biosafety, and application consistency require further evaluation. Furthermore, the stability, persistence, and uptake efficiency of dsRNA under natural mosquito-breeding-site conditions remain uncertain. Environmental factors such as temperature, ultraviolet exposure, organic matter, and microbial communities may accelerate dsRNA degradation or alter its availability to larvae. Improving dsRNA stability, formulation, and larval uptake in aquatic environments will therefore be important for translating the strong laboratory activity observed here into reliable field performance [40].

Potential non-target effects should be carefully assessed, particularly because β-tubulin is highly conserved. Sequence-based bioinformatic screening, together with empirical evaluation in relevant non-target organisms, will therefore be important before field application [41]. The potential evolution of resistance to dsRNA-based control should also be considered, as reduced dsRNA uptake, enhanced degradation, or alterations in RNAi-associated pathways have been identified as possible resistance mechanisms [42]. Formulation strategies, including nanoparticle encapsulation and other protective delivery systems, may improve dsRNA stability and uptake and thereby enhance practical performance [18]. Ultimately, semi-field and field validation will be required to determine whether laboratory efficacy can be maintained under environmentally variable conditions. The recent regulatory approval and commercialization of ledprona-based dsRNA biopesticides demonstrate the practical feasibility of RNAi-based pest control, while also emphasizing the importance of scalable production, reliable delivery, consistent field efficacy, and rigorous environmental safety assessment [43].

In conclusion, this study demonstrates that engineered bacteria-derived β-tubulin dsRNA can effectively silence target gene expression and induce high mortality in both susceptible and resistant mosquito larvae. The combined bioassay, transcriptomic, and proteomic evidence indicates that β-tubulin knockdown disrupts multiple essential biological processes, including cytoskeletal regulation, metabolism, intracellular transport, stress responses, and development. Although RNAi efficiency may vary according to mosquito species, dsRNA design, production method, and delivery conditions, the strong effects observed in both *Ae. albopictus* and *Cx. quinquefasciatus* support β-tubulin as a promising RNAi target. Further optimization of dsRNA production, formulation, aquatic delivery, and target specificity, together with standardized cross-species comparisons and field validation, will be important for translating these findings into environmentally compatible mosquito-control technologies.

## Figures and Tables

**Table 1:** Primer Sequences.

Primer	Sequence	Amplicon Size (bp)	Usage
Al β-tub F	ACCTCGAGTCGAAAACACCGACGAGACGT	365	Target fragment synthesis
Al β-tub R	ACGAGCTCGCAACTGTCAGGTAACGTCCATGT		
ClueX β-tub F	ACCTCGAGGGACAACTTTGTGTTCGGACA	362	
ClueX β-tub R	ACGAGCTCACAGGGCTTCATTGTCGAT		
dseGFP F dseGFP R	GAGCTCACTTCTTTCAAGACCCGCG CTCGAGGTAATGGTTGTCAGGCAGC	367	
qAl β-tub F	ATCGACAATGAAGCCCTGT	280	RT-qPCR analysis of β-tubulin expression
qAl β-tub R	GGAGATACGCTTGAACAGTTCCT		
qClueX β-tub F	ACTGGGCCAAGGGACACTA	249	
qClueX β-tub R	ACGGTTCCACGACGGTGTCT		
L4440 verify F	TAGGGCGAATTGGGTACCG	377	L4440 plasmid verification
L4440 verify R	GGCAGATCTGATATCATCGATGAAT		
RPL8 F	AGTTCAAGCTCCGCAAGCA	128	Reference gene for RT-qPCR normalization
RPL8 R	CACGAACTGGCCGGTGTAC		
Actin F	TCCCACACAGTCCCCATCTAC	142	
Actin R	ACGAGTAGCCACGTTCAGTCAG		

## Data Availability

All data generated or analyzed during this study are included in this published article.

## Supplementary Materials

The following supporting information can be downloaded at: https://www.mdpi.com/article/10.3390/insects17090880/s1, Supplementary Figure S1, Agarose gel electrophoresis analysis of target fragments, recombinant colonies, and bacterially expressed dsRNAs; Supplementary Sequence Alignment S1. Sequence alignment of the Aedes albopictus β-tubulin target sequence with homologous sequences from non-target species; Supplementary Sequence Alignment S2. Sequence alignment of the *Culex quinquefasciatus* β-tubulin target sequence with homologous sequences from non-target species; Figure S2. Kyoto Encyclopedia of Genes and Genomes (KEGG) and Gene Ontology (GO) enrichment analyses of differentially expressed proteins following β-tubulin dsRNA treatment in *Ae. albopictus* and *Cx. quinquefasciatus*.

## References

[1] Moyes C.L., Vontas J., Martins A.J., Ng L.C., Koou S.Y., Dusfour I., Raghavendra K., Pinto J., Corbel V., David J.P. (2017). Contemporary Status of Insecticide Resistance in the Major *Aedes* Vectors of Arboviruses Infecting Humans. PLoS Negl. Trop. Dis..

[2] Liu N. (2015). Insecticide Resistance in Mosquitoes: Impact, Mechanisms, and Research Directions. Annu. Rev. Entomol..

[3] Love R.R., Sikder J.R., Vivero R.J., Matute D.R., Schrider D.R. (2023). Strong Positive Selection in *Aedes aegypti* and the Rapid Evolution of Insecticide Resistance. Mol. Biol. Evol..

[4] Salles T.S., da Encarnação Sá-Guimarães T., de Alvarenga E.S.L., Guimarães-Ribeiro V., de Meneses M.D.F., de Castro-Salles P.F., dos Santos C.R., do Amaral Melo A.C., Soares M.R., Ferreira D.F. (2018). History, Epidemiology and Diagnostics of Dengue in the American and Brazilian Contexts: A Review. Parasites Vectors.

[5] Zulfa R., Lo W.C., Cheng P.C., Martini M., Chuang T.W. (2022). Updating the Insecticide Resistance Status of *Aedes aegypti* and *Aedes albopictus* in Asia: A Systematic Review and Meta-Analysis. Trop. Med. Infect. Dis..

[6] Fire A., Xu S., Montgomery M.K., Kostas S.A., Driver S.E., Mello C.C. (1998). Potent and Specific Genetic Interference by Double-Stranded RNA in *Caenorhabditis elegans*. Nature.

[7] Setten R.L., Rossi J.J., Han S.P. (2019). The Current State and Future Directions of RNAi-Based Therapeutics. Nat. Rev. Drug Discov..

[8] Zhu K.Y., Palli S.R. (2020). Mechanisms, Applications, and Challenges of Insect RNA Interference. Annu. Rev. Entomol..

[9] Ortolá B., Daròs J.A. (2024). RNA Interference in Insects: From a Natural Mechanism of Gene Expression Regulation to a Biotechnological Crop Protection Promise. Biology.

[10] Munawar K., Alahmed A.M., Khalil S.M.S. (2020). Delivery Methods for RNAi in Mosquito Larvae. J. Insect Sci..

[11] Wang Z.G., Qin C.Y., Chen Y., Yu X.Y., Chen R.Y., Niu J., Wang J.J. (2024). Fusion dsRNA Designs Incorporating Multiple Target Sequences Can Enhance the Aphid Control Capacity of an RNAi-Based Strategy. Pest Manag. Sci..

[12] Zhang R., An S., Zhang Z. (2024). Chitin Synthase Genes of *Aedes albopictus* and Their Effects on Development of Pupae. Arch. Insect Biochem. Physiol..

[13] Siddiqui J.A., Fan R., Naz H., Bamisile B.S., Hafeez M., Ghani M.I., Wei Y., Xu Y., Chen X. (2023). Insights into Insecticide-Resistance Mechanisms in Invasive Species: Challenges and Control Strategies. Front. Physiol..

[14] Hough J., Howard J.D., Brown S., Portwood D.E., Kilby P.M., Dickman M.J. (2022). Strategies for the Production of dsRNA Biocontrols as Alternatives to Chemical Pesticides. Front. Bioeng. Biotechnol..

[15] Xu X., Yu T., Zhang D., Song H., Huang K., Wang Y., Shen L., Li Y., Wang F., Zhang S. (2023). Evaluation of the Anti-Viral Efficacy of Three Different dsRNA Nanoparticles against Potato Virus Y Using Various Delivery Methods. Ecotoxicol. Environ. Saf..

[16] Arjunan N., Thiruvengadam V., Sushil S.N. (2024). Nanoparticle-Mediated dsRNA Delivery for Precision Insect Pest Control: A Comprehensive Review. Mol. Biol. Rep..

[17] Das S., Debnath N., Cui Y., Unrine J., Palli S.R. (2015). Chitosan, Carbon Quantum Dot, and Silica Nanoparticle Mediated dsRNA Delivery for Gene Silencing in *Aedes aegypti*: A Comparative Analysis. ACS Appl. Mater. Interfaces.

[18] Dhandapani R.K., Gurusamy D., Howell J.L., Palli S.R. (2019). Development of CS-TPP-dsRNA Nanoparticles to Enhance RNAi Efficiency in the Yellow Fever Mosquito, *Aedes aegypti*. Sci. Rep..

[19] Mysore K., Hapairai L.K., Wei N., Realey J.S., Scheel N.D., Severson D.W., Duman-Scheel M. (2019). Preparation and Use of a Yeast shRNA Delivery System for Gene Silencing in Mosquito Larvae. Methods Mol. Biol..

[20] Ding J., Cui C., Wang G., Wei G., Bai L., Li Y., Sun P., Dong L., Liu Z., Yun J. (2023). Engineered Gut Symbiotic Bacterium-Mediated RNAi for Effective Control of *Anopheles* Mosquito Larvae. Microbiol. Spectr..

[21] Timmons L., Court D.L., Fire A. (2001). Ingestion of Bacterially Expressed dsRNAs Can Produce Specific and Potent Genetic Interference in *Caenorhabditis elegans*. Gene.

[22] Taracena M., Hunt C., Pennington P., Andrew D., Jacobs-Lorena M., Dotson E., Wells M. (2022). Effective Oral RNA Interference (RNAi) Administration to Adult *Anopheles gambiae* Mosquitoes. J. Vis. Exp..

[23] Janke C., Magiera M.M. (2020). The Tubulin Code and Its Role in Controlling Microtubule Properties and Functions. Nat. Rev. Mol. Cell Biol..

[24] Bera A., Gupta M.L. (2022). Microtubules in Microorganisms: How Tubulin Isotypes Contribute to Diverse Cytoskeletal Functions. Front. Cell Dev. Biol..

[25] Singh A.D., Wong S., Ryan C.P., Whyard S. (2013). Oral Delivery of Double-Stranded RNA in Larvae of the Yellow Fever Mosquito, *Aedes aegypti*: Implications for Pest Mosquito Control. J. Insect Sci..

[26] (2011). Test Methods of Mosquito Resistance to Insecticides—Bioassay Methods.

[27] Akhmanova A., Kapitein L.C. (2022). Mechanisms of Microtubule Organization in Differentiated Animal Cells. Nat. Rev. Mol. Cell Biol..

[28] McKenna E.D., Sarbanes S.W., Cummings S.W., Roll-Mecak A. (2023). The Tubulin Code, from Molecules to Health and Disease. Annu. Rev. Cell Dev. Biol..

[29] Figueiredo Prates L.H., Fiebig J., Schlosser H., Liapi E., Rehling T., Lutrat C., Bouyer J., Sun Q., Wen H., Xi Z. (2024). Challenges of Robust RNAi-Mediated Gene Silencing in *Aedes* Mosquitoes. Int. J. Mol. Sci..

[30] Yu N., Christiaens O., Liu J., Niu J., Cappelle K., Caccia S., Huvenne H., Smagghe G. (2013). Delivery of dsRNA for RNAi in Insects: An Overview and Future Directions. Insect Sci..

[31] Singh I.K., Singh S., Mogilicherla K., Shukla J.N., Palli S.R. (2017). Comparative Analysis of Double-Stranded RNA Degradation and Processing in Insects. Sci. Rep..

[32] Scott J.G., Michel K., Bartholomay L.C., Siegfried B.D., Hunter W.B., Smagghe G., Zhu K.Y., Douglas A.E. (2013). Towards the Elements of Successful Insect RNAi. J. Insect Physiol..

[33] Silver K. (2021). Strategies for Enhancing the Efficiency of RNA Interference in Insects. Pest Manag. Sci..

[34] Whyard S., Singh A.D., Wong S. (2009). Ingested Double-Stranded RNAs Can Act as Species-Specific Insecticides. Insect Biochem. Mol. Biol..

[35] Gong Y., Li T., Zhang L., Gao X., Liu N. (2022). The Central Role of Multiple P450 Genes and Their Co-Upregulation in Mediating Permethrin Resistance in *Culex quinquefasciatus*. Front. Physiol..

[36] Huvenne H., Smagghe G. (2010). Mechanisms of dsRNA Uptake in Insects and Potential of RNAi for Pest Control: A Review. J. Insect Physiol..

[37] Mendoza-Alatorre M., Julian-Chávez B., Solano-Ornelas S., Siqueiros-Cendón T.S., Torres-Castillo J.A., Sinagawa-García S.R., Abraham-Juárez M.J., González-Barriga C.D., Rascón-Cruz Q., Siañez-Estrada L.I. (2025). RNAi in Pest Control: Critical Factors Affecting dsRNA Efficacy. Insects.

[38] Lee M.V., Topper S.E., Hubler S.L., Hose J., Wenger C.D., Coon J.J., Gasch A.P. (2011). A Dynamic Model of Proteome Changes Reveals New Roles for Transcript Alteration in Yeast. Mol. Syst. Biol..

[39] Whyard S., Erdelyan C.N.G., Partridge A.L., Singh A.D., Beebe N.W., Capina R. (2015). Silencing the Buzz: A New Approach to Population Suppression of Mosquitoes by Feeding Larvae Double-Stranded RNAs. Parasites Vectors.

[40] Fischer J.R., Zapata F., Dubelman S., Mueller G.M., Uffman J.P., Jiang C., Jensen P.D., Levine S.L. (2017). Aquatic Fate of a Double-Stranded RNA in a Sediment–Water System Following an Over-Water Application. Environ. Toxicol. Chem..

[41] Raybould A., Burns A. (2020). Problem Formulation for Off-Target Effects of Externally Applied Double-Stranded RNA-Based Products for Pest Control. Front. Plant Sci..

[42] Khajuria C., Ivashuta S., Wiggins E., Flagel L., Moar W., Pleau M., Miller K., Zhang Y., Ramaseshadri P., Jiang C. (2018). Development and Characterization of the First dsRNA-Resistant Insect Population from Western Corn Rootworm, *Diabrotica virgifera virgifera* LeConte. PLoS ONE.

[43] Li Z., Liu Y., Liang Y., Pan T., Liu J. (2026). RNA Interference-Based Pesticides: Mechanism, Application, and Commercialization in Sustainable Pest Management. Pestic. Biochem. Physiol..

