# Supplementary material for: Engineered Escherichia coli-Derived dsRNA Identifies β-Tubulin as a Candidate RNAi Target in Mosquito Larvae
Source: Insects. 2026 Aug 23;17(9):880. doi: 10.3390/insects17090880 (PMC13607943; doi:10.3390/insects17090880)
Supplement: Supplementary file 1 [file insects-17-00880-s001.zip › insects-4454077-supplementary.pdf]

Supporting Information：

Engineered *Escherichia coli*-Derived dsRNA Identifies β-Tubulin as a Candidate RNAi Target in Mosquito Larvae

Kai Wang [1,2,†], Zhongdan Bi [2,†], Teng Zhao [2,†], Yuxi Pan [1,2], Jing Wu [2,4], Xiaohui Liu [2], Dan Xing [2], Jiahong Wu [3,*], and Chunxiao Li [2,*]

1. School of Public Health, the key Laboratory of Environmental Pollution Monitoring and Disease Control, Ministry of Education, Guizhou Medical University, Guiyang 561113, China

2. State Key Laboratory of Pathogen and Biosecurity, Beijing 100071, China

3. School of Basic Medicine Sciences, Characteristic Key Laboratory of Modern Pathogen Biology, Guizhou Medical University, Guian new Area 561113, China

4. Hunan Provincial Key Laboratory of Animal Intestinal Function and Regulation, College of Life Sciences, Hunan Normal University, Changsha 410081, China

[*] Correspondence: jiahongwu2008@hotmail.com (J.W.); lichunxiao@bmi.ac.cn (C.L.)

[†] These authors contributed equally to this work.

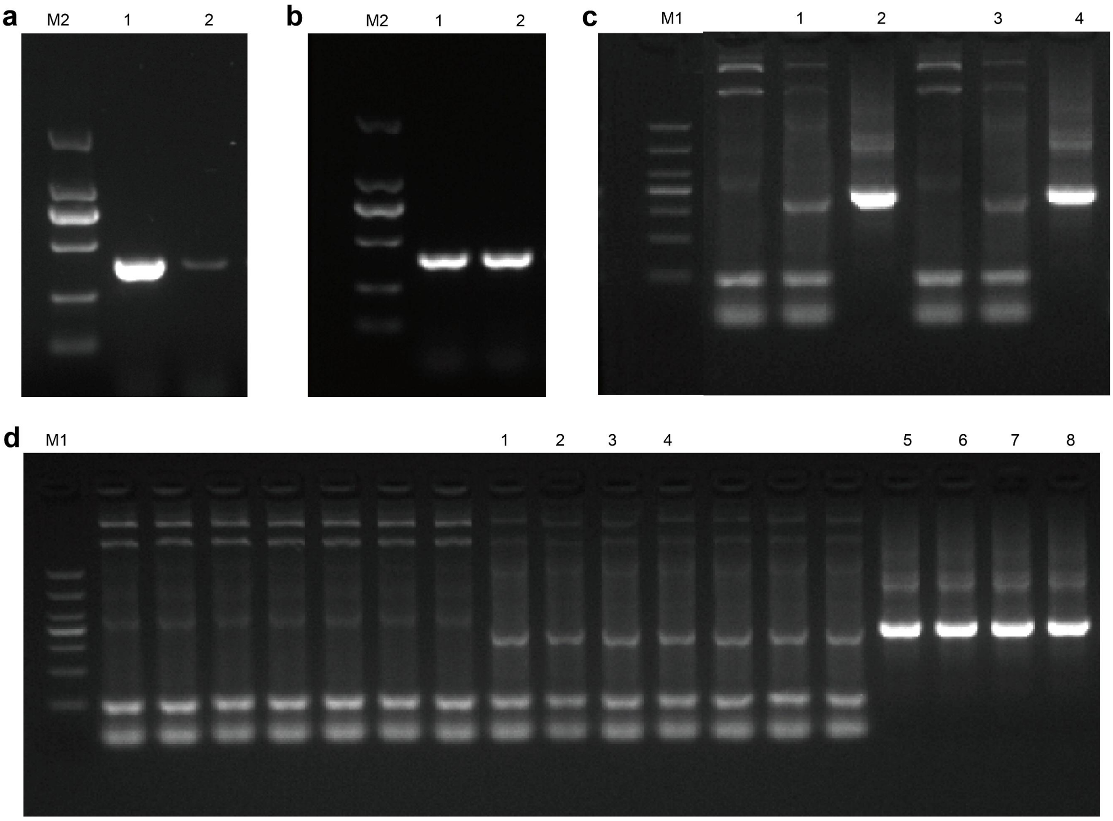

Figure S1. Agarose gel electrophoresis analysis of target fragments, recombinant colonies, and bacterially expressed dsRNAs.

a. PCR amplification of the target fragment of dseGFP. **b.** Colony PCR verification of recombinant E. coli carrying the dseGFP construct. **c.** Agarose gel electrophoresis of dseGFP dsRNA extracted from induced E. coli. Lanes 1 and 3, samples untreated with DNase I and RNase A; lanes 2 and 4, corresponding samples after DNase I and RNase A treatment. **d.** Agarose gel electrophoresis of β-tubulin dsRNA extracted from induced E. coli. Lanes 1 and 2, Culex quinquefasciatus β-tubulin dsRNA samples untreated with DNase I and RNase A; lanes 3 and 4, Aedes albopictus β-tubulin dsRNA samples untreated with DNase I and RNase A; lanes 5 and 6, C. quinquefasciatus β-tubulin dsRNA samples after DNase I and RNase A treatment; lanes 7 and 8, A. albopictus β-tubulin dsRNA samples after DNase I and RNase A treatment. M1: 1,000 bp DNA marker (1,000, 700, 500, 400, 300, 200, and 100 bp); M2: 2,000 bp DNA marker (2,000, 1000, 750, 500, 250, and 100 bp).

```
NM_131327.1        ATGCGTGAGATCGTCCACATCCAGGCCGGCAACTGGGCCAAGGGCTTCAAGATCGGCGAGATCTTCGCCA  70
GDIP01026603.1     ATGCGTGAATGTATCTCAATCCATGTCGG-----------------TCAAG-CCGG--------------  38
XM_396338.6        ATGCGTGAGATCGTCCACATCCAGGCCGGCAACTGGGCCAAGGGCTTCAAGATCGGCGAGATCTTCGCCA  70
XM_069308548.1     ----------------------------------------------------------------------  0
Ae.albopictus      ----------------------------------------------------------------------  0

NM_131327.1        AGACCTTCGACAAGTTCAAGTCGGAGATCGGCGCCAAGGTGTGGCCGGACCGCTTCAAGGTGGACTGCAA  140
GDIP01026603.1     ------------AGTCCAGATCGGAAA----CGCC---------------TGCTGGGAGTTGTATTGCC-  76
XM_396338.6        AGACCTTCGACAAGTTCAAGTCGGAGATCGGCGCCAAGGTGTGGCCGGACCGCTTCAAGGTGGACTGCAA  140
XM_069308548.1     -------------ATGGAGGCCAAGAA----------------------------AGAGAGGGACTGTAA  29
Ae.albopictus      -------------------GTCGAAAA----CACC---------------GAC---GAGACGTACTGTA-  28

NM_131327.1        GTCGGCCATCCAGGAGTGGATCGACAAGATGGCCGCTTACTACGTCGAGTGCAAGGACGTGAACGAGCTG  210
GDIP01026603.1     -TTGAACAT---GGAAT---CCAGCCTGATGGCCAAATGCCA--TC-------------TGATAAGTCAG  124
XM_396338.6        GTCGGCCATCCAGGAGTGGATCGACAAGATGGCCGCTTACTACGTCGAGTGCAAGGACGTGAACGAGCTG  210
XM_069308548.1     CTTTGTGA------------CCGACCTCACG--------CTGGGTCT------------TG---------  58
Ae.albopictus      -TCGACAAT---GAAGC---CCTGTATGAT-----------A--TC-------------TG---------  56

NM_131327.1        CAGGACATCAAGGAGTCCATCTCGCACATCGGCGAGAAGATCTGGTTGGACCTCGAGTCCATGGCCATGG  280
GDIP01026603.1     TTGGAGGTGGTGACGACTCTTTC-AACACCTTC--------TTCTCTGAAACAGGTTCTGGCAAGCACGT  185
XM_396338.6        CAGGACATCAAGGAGTCCATCTCGCACATCGGCGAGAAGATCTGGTTGGACCTCGAGTCCATGGCCATGG  280
XM_069308548.1     --------------------TCGATACACT---------------TAGAGGCGTGGG-----GGGCGTGC  88
Ae.albopictus      -------------------CTTC-CGCACC---------------CTGAAGCT------------CACAA  79

NM_131327.1        AAGTCCGCGGCAACAAGATCGCCTACATCCGCGAGTGCAAGCTGCGCGACC-TGAAGAACGTGGACTCCG  349
GDIP01026603.1     ---CCCTCG----------TGCTGTCTTTGTCGACTTGGAAC----CAACCGTCGTTGATGAGGTTCGCA  238
XM_396338.6        AAGTCCGCGGCAACAAGATCGCCTACATCCGCGAGTGCAAGCTGCGCGACC-TGAAGAACGTGGACTCCG  349
XM_069308548.1     AG---------------------------------------------GACG-------------------  94
Ae.albopictus      ---CCC----------------------------------------CAACC-------------------  87

NM_131327.1        TGGTCGCCGAGTACGA----CGAGTCCGGCGAGACCTACTGCGAGATGAACGACGTCCACTCCTTCGTCT  415
GDIP01026603.1     CTGGCACT---TACAGATCCCTGTTTCACCCTGAGCAGCTG----ATTACTGGCAAGGAAGATGCCGCTA  301
XM_396338.6        TGGTCGCCGAGTACGA----CGAGTCCGGCGAGACCTACTGCGAGATGAACGACGTCCACTCCTTCGTCT  415
XM_069308548.1     -------TGAGTGTGG-----------------------TG----GTGCGTGGGG---------------  115
Ae.albopictus      -----------TACGG-----------------------TG----AT--CTG------------------  99

NM_131327.1        GGAAGTTCGCCAAGGTCCGCGAGTACGACATCTCCAAGAAGGCCATCAACATCGTGCC--------TCCG  477
GDIP01026603.1     ATAACTATGCCCGTGGCCAC---TACACTATTGGCAAGGAAATCGTTGATCTGGTGTTGGACCGTGTCCG  368
XM_396338.6        GGAAGTTCGCCAAGGTCCGCGAGTACGACATCTCCAAGAAGGCCATCAACATCGTGCC--------TCCG  477
XM_069308548.1     ---------------------------------GCGTGGAGCGCGCCTCCGTGGTGGC------------  140
Ae.albopictus      ----------------------------------------AACC----ATCTGGTGT-------------  112

NM_131327.1        CCGGTGGTCACCCAGGAGTACCGCGACGCCGTCTACGTCAACTCGGCCACCGTGCTGCCGGAGGAG-CAC  546
GDIP01026603.1     CAAGCTCTCCGATCAATGCACTG----GTCTTCAGGGATTCCTCATTTTCCACTCTTTCGGTGGAGGCAC  434
```

XM_396338.6 CCGGTGGTCACCCAGGAGTACCGCGACGCCGTCTACGTCAACTCGGCCACCGTGCTGCCGGAGGAG-CAC 546
XM_069308548.1 -----------GTGGGAGCAGCGGCACAACGTA------------------GCCCTGCCGGCGG------ 175
Ae. albopictus ------------------CACTG----ACC-- 120

NM_131327.1 GGCATCGA----CAAGATCGTGCACGCCAAGCGCGCCGTGGTCGACCTCGGCGACCGCATCAACGTGGAC 612
GDIP01026603.1 TGGCTCGGGTTTCACCTCCCTGCTTATGGAACGTCTCTCAGTTGACTACGGCAAGAAATCCAAGTTGGA- 503
XM_396338.6 GGCATCGA----CAAGATCGTGCACGCCAAGCGCGCCGTGGTCGACCTCGGCGACCGCATCAACGTGGAC 612
XM_069308548.1 ------------------------CACTCAGGGCCCTCTA-----------------CACCGCCACCGAC 204
Ae. albopictus ------------------------------ATGTCC------------------------------GGA- 129

NM_131327.1 GTCCGCGAGGCCCTGGTCCGCGGCAAGATCCACAAGT-TCGTCGAGTTGTCCGACTGGCCCTCCGGAAAG 681
GDIP01026603.1 GTTCGCCATTTACCCAGCCCCGCAAGTCTCCACTGCTGTTGTTGAACCTTACAACT--CCATTTTGA--- 568
XM_396338.6 GTCCGCGAGGCCCTGGTCCGCGGCAAGATCCACAAGT-TCGTCGAGTTGTCCGACTGGCCCTCCGGAAAG 681
XM_069308548.1 GGCTTCAAACT---CACCTGGAACTA 227
Ae. albopictus GT---AA--- 133

NM_131327.1 TTCGACAAGTCGAAGATGGGCGTGTTCCTGTCGGTCGACTCGGTCGTCGAGACCAACGAGGCGTTCTGCC 751
GDIP01026603.1 --CCACCCATACTACTTTGGAACATTCCGATTG--CGCTTTCATGGTCGA---TAACGAAGCTATTTA-- 629
XM_396338.6 TTCGACAAGTCGAAGATGGGCGTGTTCCTGTCGGTCGACTCGGTCGTCGAGACCAACGAGGCGTTCTGCC 751
XM_069308548.1 CGCCACAG------------------CAGGGAAGGTGCTGCCACTGGGGAA--CA--------------- 262
Ae. albopictus --CCAC--------------------CTGCCTG--CGATTCCCTGGTCAA---C---------------- 160

NM_131327.1 GCGGCATCTACTCGGCCATGAAGGTCGCC--GTCACCTCGCTGCGCAAGTTCGACCTGACCGAGCGCATG 819
GDIP01026603.1 -TGACATTTGTCGTCGCAATCTAGACATCGAGCGTCCTAGCTACACCAATTTGAATCGTCTGATTG---G 695
XM_396338.6 GCGGCATCTACTCGGCCATGAAGGTCGCC--GTCACCTCGCTGCGCAAGTTCGACCTGACCGAGCGCATG 819
XM_069308548.1 -TGG----------------------------------AGATCCACCCACTGGG---------------- 281
Ae. albopictus -TGA--------------------------------------ACGCCGATCTG----------------- 174

NM_131327.1 ACCGAGGTGCTGCGCGAGTACAAGGACTCGTCCAACAAGTGGATCGA-GTCGTTCAAGGCCAAGGAGAAG 888
GDIP01026603.1 TCAGATCGTCTCTTCCATTACAGCTTCTCTTC--------GCTTCGATGGCGCATTGAACGTCG----AT 753
XM_396338.6 ACCGAGGTGCTGCGCGAGTACAAGGACTCGTCCAACAAGTGGATCGA-GTCGTTCAAGGCCAAGGAGAAG 888
XM_069308548.1 --------------------------------------GAGGGTCAA--------CAGGCTGGG----GA 301
Ae. albopictus --CGA---------AAAC---------- 181

NM_131327.1 GAGGCCGAGAACTTCACCGCGTGGGTGGACTCCGTCCGCCGCTTCGACAAGGCCAAGTTCATGGAGTCCA 958
GDIP01026603.1 TTGACCGAA---TTCCAGACGAACTTGGTCCCTTACCCTCGTATCCAT---------TTC-------CCA 804
XM_396338.6 GAGGCCGAGAACTTCACCGCGTGGGTGGACTCCGTCCGCCGCTTCGACAAGGCCAAGTTCATGGAGTCCA 958
XM_069308548.1 GTGGGAGGGG----------GAGCGGAGACCCCCTCGCCC-CCTCCAT---------CAC---------- 341
Ae. albopictus -TGGCCG------TC------AACATGGTTCCCTTCCCACGTCTGCAC---------TTC-------T-- 220

NM_131327.1 TCCGCGAGTCGTGC--GAGTT-CATCCGCGACATGGCCAAGGAGTACGTCCGCGAGGGCGAGAAGTGGAT
1025
GDIP01026603.1 TTGGTAACTTATGCTCCAGTTATTTCCGCTG--------AAAAGGCTTACCATGAGC-----AGTTGACA 861
XM_396338.6 TCCGCGAGTCGTGC--GAGTT-CATCCGCGACATGGCCAAGGAGTACGTCCGCGAGGGCGAGAAGTGGAT

1025
XM_069308548.1 ----CGACCTG-GCCCTGGCT--------------------GAGGACCCCC-----------------CT 370
Ae. albopictus --------TCATGCCCGGATT-----CGC--------------------CC-----------------CA 240

NM_131327.1 CTCGGAGGAGGACGTGGAGGACTTCAAGGCCAAGGTCCGC--GAGGCCATC--GCCGAGGAGTGGGGCGA
1091
GDIP01026603.1 GTCGGAGAGATC-----ACGACCTC-----------CTGCTTTGAACCAGCCAACCAAATGGTGAAATGC 915
XM_396338.6 CTCGGAGGAGGACGTGGAGGACTTCAAGGCCAAGGTCCGC--GAGGCCATC--GCCGAGGAGTGGGGCGA
1091
XM_069308548.1 CTCCTGCAGAAC--------ACCCC-----------CAGC------------------------------ 391
Ae. albopictus CTC-----------------ACCTC-----------CCGC------------------------------ 252

NM_131327.1 GGCGATGGACATGGCCAAGGACGCCGTGGCCCACTTCGTGGAGTCGATGGAGAACGTGAAGTCGTA--CG
1159
GDIP01026603.1 GATCCTCGTCATGGCAAATA----CATGGCCTGCTGCATGTTGT--------ATCGCGGTGACGTAGTTC 973
XM_396338.6 GGCGATGGACATGGCCAAGGACGCCGTGGCCCACTTCGTGGAGTCGATGGAGAACGTGAAGTCGTA--CG
1159
XM_069308548.1 --------------------------------ACCGCCCAGGGG-------GGTCACGACCCC------- 415
Ae. albopictus --GG--------ATCGCAA----------- 261

NM_131327.1 AGGACGAGATCGGCGCCGA---GGCCAAGCAGAAGGAGTCGGAGTTTGCCA---AGGCCATCAACACCGT
1223
GDIP01026603.1 CCAAAGATGTCAATGCTGCTATTGCCACCATTAAAA---CTAAACGTTCTATCCAGTTCGTTGATTGGTG
1040
XM_396338.6 AGGACGAGATCGGCGCCGA---GGCCAAGCAGAAGGAGTCGGAGTTTGCCA---AGGCCATCAACACCGT
1223
XM_069308548.1 ----------CACTCACGC---CGCC-------------CCCAACCTTCCA------------------- 440
Ae. albopictus ----------CAGTACCG----TGCC-------------CTGACCGTGCCA-----------GA------ 287

NM_131327.1 CTTCGACGGCAAGGAGATCAACTCCGCCATCGACGCCATTCGCAAGTGGCTGCACTCCGGCTTCGACGTC
1293
GDIP01026603.1 CCCTACTGGTTTTAAGGTTGGTATCAAC-----TACCAGCCACCAACTGTTGT-CCCTGGTGGTGATCTC
1104
XM_396338.6 CTTCGACGGCAAGGAGATCAACTCCGCCATCGACGCCATTCGCAAGTGGCTGCACTCCGGCTTCGACGTC
1293
XM_069308548.1 -----CTCCT--------------------------------CTAA--GATGT-TCGAGGTG--GACAGC 468
Ae. albopictus ----ACTGAC--------------------------------CCAACAGATGT-TC--------GA---C 309

NM_131327.1 TGCTTCAAGGTGGCCATGGTCGCCGGCGACACCGTGGCCGAGATGGAGCGCTGCAAGTGGCTGCGCAAGG
1363
GDIP01026603.1 -GC--CAAGGTGTCCCG---TGCTGTTTGCATGTTGTCCAACAC----TACTGCTATCGCTGAAGC----
1160
XM_396338.6 TGCTTCAAGGTGGCCATGGTCGCCGGCGACACCGTGGCCGAGATGGAGCGCTGCAAGTGGCTGCGCAAGG
1363

XM_069308548.1 TGT--CAAGG---------------------CTTTGGT------------------------AAGT---- 487
Ae. albopictus -GC--CAAGA------------------ACATGATGGCC-------------------------GC---- 329

NM_131327.1 AGCACACCGGCGCCAAGTGGTTCCAGG---TCATCTCGGGCGAGGCCATCCCGCACCGGGCATCGTCCA 1430
GDIP01026603.1 ------CTGGGCTCGTTTGGATCACAAGTTTGACTTGATGTATGCTAAACGTGCTTTTGTTCACTGGT-A 1223
XM_396338.6 AGCACACCGGCGCCAAGTGGTTCCAGG---TCATCTCGGGCGAGGCCATCCCGCACCGGGCATCGTCCA 1430
XM_069308548.1 ----TTCTGGTTCAA---------------TCACCT------------------TTCAACCATTTAGCG 519
Ae. albopictus ------CTG---------------------CGACC--------------------------CAC------ 340

NM_131327.1 CACCGAGGTGGCCGTGTGCCGCATCAAGGACGCCAAGTTCGGCAACAAGCAGTTCATCGGCGA-CATCGT 1499
GDIP01026603.1 CGTTGGAGAGG---------GCATGGAGGAAGGAGAGTTCTCCGAAGCCCGTGAAGATCTCGC-TGCTCT 1283
XM_396338.6 CACCGAGGTGGCCGTGTGCCGCATCAAGGACGCCAAGTTCGGCAACAAGCAGTTCATCGGCGA-CATCGT 1499
XM_069308548.1 CACGTTAGAGGCT-CGCTCCGTGTGGGCTTCAGTGAG----------------------GTGTATGTCAA 566
Ae. albopictus ----------G---------ACATGGA---------------------------------CGT-TACCTG 357

NM_131327.1 GGAGCCG---TCCAAGGTGGTCGACCGCATCCGCGCCGCCGTCAACGCGTGGATCCGCGAGCAGCACAAG 1566
GDIP01026603.1 AGAGAAGGATTACGAAGAGGTTG---GTATGGATTCCGTCGAGGGAGAGGGAGAAGGCGGTGATGAATAT 1350
XM_396338.6 GGAGCCG---TCCAAGGTGGTCGACCGCATCCGCGCCGCCGTCAACGCGTGGATCCGCGAGCAGCACAAG 1566
XM_069308548.1 GGAGGAGGTGTACTCGGCGACCC------TCAGGTGTGAAGTGCAAGCATGGCTGTGTGCTTGA------ 624
Ae. albopictus ACAG-----TTGC--- 365

NM_131327.1 TAA 1569
GDIP01026603.1 TAA 1353
XM_396338.6 TAA 1569
XM_069308548.1 --- 624
Ae. albopictus --- 365

Supplementary Sequence Alignment S1. Sequence alignment of the *Aedes albopictus* β-tubulin target sequence with homologous sequences from non-target species.

The β-tubulin target sequence of *Aedes albopictus* was aligned with homologous sequences from four non-target species: *Danio rerio* (NM_131327.1), *Daphnia magna* (GDIP01026603.1), *Apis mellifera* (XM_396338.6), and *Procambarus clarkii* (XM_069308548.1). The *Ae. albopictus* sequence shown in the alignment represents the target sequence used for comparison.

NM_131327.1 ATGCGTGAGATCGTCCACATCCAGGCCGGCAACTGGGCCAAGGGCTTCAAGATCGGCGAGATCTTCGCCA
70
GDIP01026603.1 ATGCGTGAATGTATCTCAATCCATGTCGGT---CAAGCCGGAG---TCCAGATCGG--AAACGCCTGCTG
62
XM_396338.6 ATGCGTGAGATCGTCCACATCCAGGCCGGCAACTGGGCCAAGGGCTTCAAGATCGGCGAGATCTTCGCCA
70
XM_069308548.1 --
0
Cx. quinquefasciatus --
0

NM_131327.1 AGACCTTCGACAAGTTCAAGTCGGAGATCGGCGCCAAGGTGTGGCCGGACCGCTTCAAGGTGGACTGCAA
140
GDIP01026603.1 GGAGTTGTATTGCCTTGAACATGGAATCCAGCCTGA-----TGGCCAAATGCCATCTGA-----------
116
XM_396338.6 AGACCTTCGACAAGTTCAAGTCGGAGATCGGCGCCAAGGTGTGGCCGGACCGCTTCAAGGTGGACTGCAA
140
XM_069308548.1 --
0
Cx. quinquefasciatus --
0

NM_131327.1 GTCGGCCATCCAGGAGTGGATCGAC---AAGATGGCCGCTTACTACGTCGAGTGCAAGGACGTGAACGAG
207
GDIP01026603.1 -TAAGTCAGTTGGAGGTGG--TGAC---GA------CTCTTTCAACACCTTCTTCTCTGA----AACAGG
170
XM_396338.6 GTCGGCCATCCAGGAGTGGATCGAC---AAGATGGCCGCTTACTACGTCGAGTGCAAGGACGTGAACGAG
207
XM_069308548.1 -ATGGAGGCCAAGAAAGAGAGGGACTGTAA------CTTT-----------------------------G
34
Cx. quinquefasciatus ---------------------GGAC---AA------CTTTGT---------------------------G
13

NM_131327.1 CTGCAGGACATCAAGGAGTCCATCTCGCACATCGGCGAGAAGATCTGGTTGGACCTCGAGTCCATGGCCA

277

GDIP01026603.1 TT-CTGG----CAAGCA---CGTCCCTCGTGCTGTC--------TTTGTCG-ACTTGGAACCAACCGTCG

223

XM_396338.6 CTGCAGGACATCAAGGAGTCCATCTCGCACATCGGCGAGAAGATCTGGTTGGACCTCGAGTCCATGGCCA

277

XM_069308548.1 TGACCGACC-TCA---------------------------------CGCTGGTCT---------------

56

Cx. quinquefasciatus TT-CGGA----CA----------------------------------GTCG-G-----------------

26

NM_131327.1 TGGA---AGTCCGCGGCAACAAGATCGCCTACAT-CCGCGAGTGCAAGCTGCGCGACC--TGAAGAACGT

341

GDIP01026603.1 TTGATGAGGTTCGCACTGGCA------CTTACAGATCCCTGTTTCACCCTGAGCAGCTGATTACTGGCAA

287

XM_396338.6 TGGA---AGTCCGCGGCAACAAGATCGCCTACAT-CCGCGAGTGCAAGCTGCGCGACC--TGAAGAACGT

341

XM_069308548.1 -------------TGTCGATA----CACTTAGAG---------GCGTGGGGGGCG-----TGCAGGACGT

95

Cx. quinquefasciatus ------------GCGCCGGCA---------ACAA--------------CTGGGC-------------CAA

48

NM_131327.1 GGACTCCGTGGTCGCCGAGTACGACGAGTCCGGCGAGACCTACTGCGAGATGAACGACGTCCACTCCTTC

411

GDIP01026603.1 GGA------AGATGCCGCTAATAACTATGCCCGTGG---CCACTACACTATTGGCAAGGA--AATCGTTG

346

XM_396338.6 GGACTCCGTGGTCGCCGAGTACGACGAGTCCGGCGAGACCTACTGCGAGATGAACGACGTCCACTCCTTC

411

XM_069308548.1 GAGT---GTGG-----------------------------TGGTGCGTGG-GGGCGTGGAGCGCGCCTCC

132

Cx. quinquefasciatus GGG------A------------------------------CACTACACCGAGGGCGCGGA--ACTGGTCG

80

NM_131327.1 GTCTGGAAGTTCGCCAAGGTCCGCGAGTACGACATCTCCAAGAAGGCCATCAACATCGTGCCTCCGCCGG

481

GDIP01026603.1 ATCTGGT-GTTGGACCGTGTCCGCAAGC------TCTCCGA-------------TCAATGCACTGGTC--

394

XM_396338.6 GTCTGGAAGTTCGCCAAGGTCCGCGAGTACGACATCTCCAAGAAGGCCATCAACATCGTGCCTCCGCCGG

481

XM_069308548.1 GTGGTGGCGTGGGAGCAGCGGCACAACG-TAGCCCTGCCGGCGGCACTCAGGGCCCTCTAC-ACCGCC--

198

Cx. quinquefasciatus ACAGCGT-GCTCGACGTGGTGCGCAAGG------AGGCCGA-------------ATCGTGCGACTGCC--

128

NM_131327.1 TGGTCACCCAGGAGTACCGCGACGCCGTCT---ACGTCAACTCGGCCACCGTGCTGCCGGAGGAGCACGG

548

GDIP01026603.1 ------TTCAGGGATTCC---TCATTTTCC---AC-TCT-TTCGGT------------GGAGGCAC-TGG

437

XM_396338.6 TGGTCACCCAGGAGTACCGCGACGCCGTCT---ACGTCAACTCGGCCACCGTGCTGCCGGAGGAGCACGG

548

XM_069308548.1 -----ACCGACGGCTTCA---AACTCACCTGGAACTACGCCACAGCAGG---------GAAGGTGC-TGC

250

Cx. quinquefasciatus ------TGCAGGGATTCC---AGCTGACGC---AC-TCG-CTGGGC------------GGTGGTAC-CGG

171

NM_131327.1 CATCGA---CAAGATCGTGCACGCCAAGCGCGCCGTGGTCGACCTCGGCGACCGCATCAACGTGGACGTC

615

GDIP01026603.1 CTCGGGTTTCACCTCCCTGCTTATGGAACGTCTCTCAGTTGACTACGGCAAG-AAATCCAAGTTGGAGTT

506

XM_396338.6 CATCGA---CAAGATCGTGCACGCCAAGCGCGCCGTGGTCGACCTCGGCGACCGCATCAACGTGGACGTC

615

XM_069308548.1 CACTGG-----GGAACATG---GAGATCCACCCACTGGGGAGGGTCAACAGGCTGGGGAGTGGGAGGGGG

312

Cx. quinquefasciatus TTCCGG---------CATG-----GGAACG--

187

NM_131327.1 CGCGAGGCCCTGGTCCGCGGCAAGATCCACAAGTTCGTCGAGTTGTCCGACTGGCCCTCCGGAA---AGT

682

GDIP01026603.1 CGCCA----TTTACCC------AGCCCCGCAAGTCTCCACTGCTGTT--GTTGAACCTTACAACTCCATT

564

XM_396338.6 CGCGAGGCCCTGGTCCGCGGCAAGATCCACAAGTTCGTCGAGTTGTCCGACTGGCCCTCCGGAA---AGT

682

XM_069308548.1 AGCGGAG-----------------------------ACCCCCTCGCC-------CCCTCCATCA------

340

Cx. quinquefasciatus ---------------------------------------CTGCTG---------ATCTCA----------

199

NM_131327.1 TCGACAAGTCGAAGA---TGGGCGTGTTCCTGTCGGTCGACTCGGTCGTCGAGACCAACGAGGC-GTTCT

748

GDIP01026603.1 TTGACCACCCATACTACTTTGGAACATTCCGATTG--CGCTTTCATGGTCGA---TAACGAAGCTATTT-

628

XM_396338.6 TCGACAAGTCGAAGA---TGGGCGTGTTCCTGTCGGTCGACTCGGTCGTCGAGACCAACGAGGC-GTTCT

748

XM_069308548.1 --CCGACCTGGCCC

352

Cx. quinquefasciatus --AAG--ATTC-

206

NM_131327.1 GCCGCGGCATCTACT-CGGCCATGAAGGTCGCCGTCA-CCTCGCTGCGCAAGTTCGACCTGACCGAGCGC

816

GDIP01026603.1 ---ATGACATTTGTCGTCGCAATCTAGACATCGAGCGTCCTAGCTACACCAATTTGAATCGTCTGATTG-

694

XM_396338.6 GCCGCGGCATCTACT-CGGCCATGAAGGTCGCCGTCA-CCTCGCTGCGCAAGTTCGACCTGACCGAGCGC

816

XM_069308548.1 TGGCTGAGGACCCCCCTCTCCTGCAGA------------------ACACC------------CCCAGCAC

393

Cx. quinquefasciatus ---GTGA------------------AGA-----------------ATACC------------CCGA----

222

NM_131327.1 ATGACCGAGGTGCTGCGCGAGTACAAGGACTCGTCCAACAAGTGGATCGAGTCGTTCAAGGCCAAGGAGA

886

GDIP01026603.1 --GTCAGATCGTCTCTTCCATTACAGCTTCTCTTC----GCTTCGATGGCG-CATTGAACGTCGATTTGA

757

XM_396338.6 ATGACCGAGGTGCTGCGCGAGTACAAGGACTCGTCCAACAAGTGGATCGAGTCGTTCAAGGCCAAGGAGA

886

XM_069308548.1 CGCCCAGGGG--------GGTCA--------------------------------CGACCCCC-------

416

Cx. quinquefasciatus ----CAGA-----------ATCA--------------------------------TGAAC----------

235

NM_131327.1 AGGAGGCCGAGAACTTCACCGCGTGGGTGGACTCCGTCCGCCGCTTCGACAAGGCCAAGTTCATGGAGTC

956

GDIP01026603.1 ------CCGA--ATTCCA-------GACGAACTTGGTCCCTTACCCT--------CGTATCCATTT--CC

802

XM_396338.6 AGGAGGCCGAGAACTTCACCGCGTGGGTGGACTCCGTCCGCCGCTTCGACAAGGCCAAGTTCATGGAGTC

956

XM_069308548.1 --------------------------ACTCACGCCGCCCCCAACCTT----------------------C

438

Cx. quinquefasciatus --------------------------ACATACTCAGTCG--TGCCCT--------CG-----------CC

258

NM_131327.1 CATCCGCGAGTCGTGCG--AGTTCATCCGCGACATGGCCAAGGAGTACGTCCGCGAGGGCGAGAAGTGGA

1024

GDIP01026603.1 CATTGGTAACTTATGCTCCAGTTATTTC----CGCTGAAAAGGCTTAC---CATGA------GCAGTTGA

859

XM_396338.6 CATCCGCGAGTCGTGCG--AGTTCATCCGCGACATGGCCAAGGAGTACGTCCGCGAGGGCGAGAAGTGGA

1024

XM_069308548.1 CACTCCT------------------------------CTAAGA--TGT--TCG----------AGGTGGA

464

Cx. quinquefasciatus CA-------------------------------------AAG---TGT---CA---------------GA

270

NM_131327.1 TCTCGGAGGAGGACGTGGAGGACTTCAAGGCCAAGGTCCGCGAGGCCATCGCCGAGGAGTGGGGCGAGGC

1094

GDIP01026603.1 CAGTCGGAGAGATCACG----ACCTCCTGCTTTGAACCAGCCAA-------CCAAATGGTGAAATGCGAT

918

XM_396338.6 TCTCGGAGGAGGACGTGGAGGACTTCAAGGCCAAGGTCCGCGAGGCCATCGCCGAGGAGTGGGGCGAGGC

1094

XM_069308548.1 CA--

466

Cx. quinquefasciatus CA--

272

NM_131327.1 GATGGACA------TGGCCAAGGACGCCGTGGCCCACTTCGTGGAGTCGATGGAGAACGTGAAGTCGTAC

1158

GDIP01026603.1 CCTCGTCA------TGGCAAATA----CATGGCCTGCTGCATGTTGT--------ATCGCGGTGACGTAG

970

XM_396338.6 GATGGACA------TGGCCAAGGACGCCGTGGCCCACTTCGTGGAGTCGATGGAGAACGTGAAGTCGTAC

1158

XM_069308548.1 -GCTGTCAAGGCTTTGGTAAGTTTC---------------------------------------TGGT--

494

Cx. quinquefasciatus --CCGTCG------TGG--AAC---CGTA-

288

NM_131327.1 GAGGACGAGATCGGCGCCGAGGCCAAGCAGAAGGAGTCGGAGTTTGCCAAGGCCATCAACACCGTCTTCG

1228

GDIP01026603.1 ---TTCCCAAAGATGTCAATGCTGCTATTG

997

XM_396338.6 GAGGACGAGATCGGCGCCGAGGCCAAGCAGAAGGAGTCGGAGTTTGCCAAGGCCATCAACACCGTCTTCG

1228

XM_069308548.1 ---TCAAT----------

499

Cx. quinquefasciatus --CAACGC--------

294

NM_131327.1 ACGGCAAGGAGATCAACTCCGCCATCGACGCCATTCGCAAGTGGCTGCACT-CCGGCTTCGACGTCTGCT

1297

GDIP01026603.1 CCACCATTAAAACTAAACGTTCTATC----CAGTTCGTTGATTGGTGCCCTACTGGTTTTAAGGTTGGTA

1063

XM_396338.6 ACGGCAAGGAGATCAACTCCGCCATCGACGCCATTCGCAAGTGGCTGCACT-CCGGCTTCGACGTCTGCT

1297

XM_069308548.1 -CACCT----------TTCAACCATTTA----------------GCGCACG-------------------

523

Cx. quinquefasciatus -CACC----------------CTGTC----C-------------GTGC----------------------

308

NM_131327.1 TCAA--GGTGGCCA-------TGGTCGCCG-GCGACACCGTGGCCGAGATGGA--GCGCTGCAAGTG-GC

1354

GDIP01026603.1 TCAACTACCAGCCACCAACTGTTGTCCCTG-GTGGTGATCTCGCCAAGGTGTCCCGTGCTGTTTGCATGT

1132

XM_396338.6 TCAA--GGTGGCCA-------TGGTCGCCG-GCGACACCGTGGCCGAGATGGA--GCGCTGCAAGTG-GC

1354

XM_069308548.1 TTAGAGGCTCGCT------------CCGTGTGG-------------------------------------

544

Cx. quinquefasciatus ------ACCAGC----------------TG-GTAG----------AA-----------------------

322

NM_131327.1 TGCGCAAGGAGCACACCGGCGCCAA----GTGGTTCCAGGTCATCTCGG--GCGAGGCCATCCCG-----

1413

GDIP01026603.1 TGTCCA------ACACTACTGCTATCGCTGAAGCCTGGGCTCGTTTGGATCACAAGTTTGACTTGATGTA

1196

XM_396338.6 TGCGCAAGGAGCACACCGGCGCCAA----GTGGTTCCAGGTCATCTCGG--GCGAGGCCATCCCG-----

1413

XM_069308548.1 ------------GCTTCA---------------------------------GTGAG--------------

555

Cx. quinquefasciatus -----A------ACACCG---------------------------------ACGAG--------------

334

NM_131327.1 ---CACCCGGGCATCGTCCACACCGAGGTGGCCGTGTGCCGCA-TCAAGGACGCCAAGTTCGGCAACAAG

1479

GDIP01026603.1 TGCTAAACGTGC-TTTTGTTCACTGGTACGTTGGAGAGG-GCA-TGGAGGAAGGAGAGTTCTCCGA----

1259

XM_396338.6 ---CACCCGGGCATCGTCCACACCGAGGTGGCCGTGTGCCGCA-TCAAGGACGCCAAGTTCGGCAACAAG

1479

XM_069308548.1 ------GTGTATGT------CAAGGAGGAGGT--------GTACTCGGCGACCCTCAGGT--GTGA----

599

Cx. quinquefasciatus ------ACGTAC-T--------------------------GTA-TCGACAA------------TGA----

354

NM_131327.1 CAGTTCAT--------CGGCGACATCGTGGAGCCGTCCAAGGTGGTCGACCGCATCCGCGCCGCCGTCAA

1541

GDIP01026603.1 -AGCCCGTGAAGATCTCGCTGCTCTAGAGAAGGATTACGAAGAGGTTG---GTATGGATTCCGTCGAGGG

1325

XM_396338.6 CAGTTCAT--------CGGCGACATCGTGGAGCCGTCCAAGGTGGTCGACCGCATCCGCGCCGCCGTCAA

1541

XM_069308548.1 -AGTGCAA----------GCATGGCTGTGTGCTTGA----------------------------------

624

Cx. quinquefasciatus -AGCCC-------------TGT--

362

NM_131327.1 CGCGTGGATCCGCGAGCAGCACAAGTAA 1569

GDIP01026603.1	AGAGGGAGAAGGCGGTGATGAATATTAA	1353
XM_396338.6	CGCGTGGATCCGCGAGCAGCACAAGTAA	1569
XM_069308548.1	----------------------------	624
Cx. quinquefasciatus	----------------------------	362

Supplementary Sequence Alignment S2. Sequence alignment of the *Culex quinquefasciatus* β-tubulin target sequence with homologous sequences from non-target species.

The β-tubulin target sequence of *Culex quinquefasciatus* was aligned with homologous sequences from four non-target species: *Danio rerio* (NM_131327.1), *Daphnia magna* (GDIP01026603.1), *Apis mellifera* (XM_396338.6), and *Procambarus clarkii* (XM_069308548.1). The *Cx. quinquefasciatus* sequence shown in the alignment represents the target sequence used for comparison.

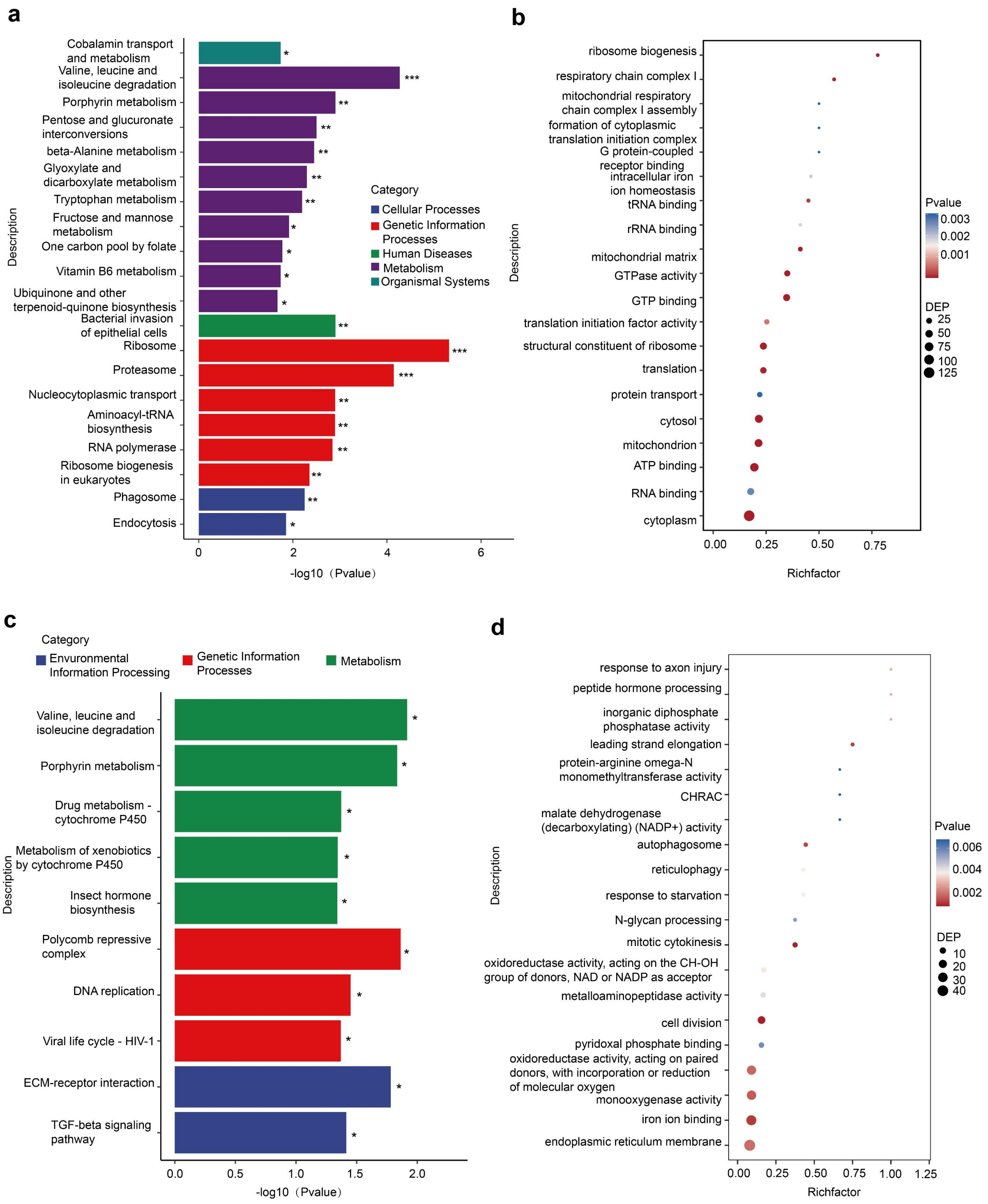

Figure S2. Kyoto Encyclopedia of Genes and Genomes (KEGG) and Gene Ontology (GO) enrichment analyses of differentially expressed proteins following β-tubulin dsRNA treatment in *Ae. albopictus* and *Cx. quinquefasciatus*.

a-b. KEGG pathway and GO enrichment analyses, respectively, of differentially expressed proteins (DEPs) between the β-tubulin dsRNA-treated and dseGFP control groups in *Ae. albopictus*. **c-d.** KEGG pathway and GO enrichment analyses, respectively, of DEPs between the β-tubulin dsRNA-treated and dseGFP control groups in *Cx. quinquefasciatus*. In the KEGG enrichment plots (a, c), the x-axis represents −log10(P value), with larger values indicating greater statistical significance of pathway enrichment; the y-axis lists the significantly enriched KEGG

pathways, and bar colors indicate the corresponding KEGG functional categories. In the GO enrichment plots (b, d), the x-axis represents the RichFactor, defined as the ratio of the number of DEPs annotated to a given GO term to the total number of proteins annotated to that term; a larger RichFactor indicates a higher degree of enrichment. The y-axis lists the enriched GO terms. Bubble size represents the number of DEPs associated with each GO term, and bubble color represents the enrichment P value, with smaller P values indicating greater statistical significance.
